# Synthesis, Physicochemical Characterization, Biological Assessment, and Molecular Docking Study of Some Metal Complexes of Alloxan and Ninhydrin as Alterdentate Ligands

**DOI:** 10.1007/s10904-023-02661-5

**Published:** 2023-04-30

**Authors:** Mamdouh S. Masoud, Galila A. Yacout, Bassant A. Abd-El-Khalek, Ahmed M. Ramadan

**Affiliations:** 1 Chemistry Department, Faculty of Science, Alexandria University, P.O. Box 426, Alexandria, 21321 Egypt; 2 Biochemistry Department, Faculty of Science, Alexandria University, P.O. Box 21511, Alexandria, Egypt

**Keywords:** Chelation patterns, Thermal analysis, Antimicrobial efficacy, Anticancer potency, DFT calculations

## Abstract

A series of transition metal complexes of alloxan monohydrate (H_2_L^1^) and ninhydrin (H_2_L^2^) have been prepared where metal ions are Fe(III), Co(II), Ni(II), Cu(II), Zr(IV), and Mo(VI). Different microanalytical techniques, spectroscopic methods, and magnetic studies were applied to assign the mode of bonding and elucidate the structure of complexes. All solid complexes are of 1:1 (M:L) stoichiometry and octahedral geometry except nickel (II) complexes exist in a tetrahedral geometry. FTIR spectral interpretation reveals that HL^1^ coordinates to the central metal ion in a bidentate ON pattern, whereas HL^2^ behaves as an alterdentate ligand through hydroxyl oxygen and carbonyl oxygen either C(1) = O or C(3) = O. The thermal behavior of some complexes was followed up to 700 °C by different techniques (TGA, DTA, and DSC) where decomposition stages progress in complicated mechanisms and are ended by the formation of metal oxide residue. Besides, biological screening involving antioxidant, antibacterial, and antifungal for ligands and some of their complexes was done. Moreover, four examined metal complexes displayed anticancer activity against hepatocellular carcinoma cells (HepG-2) but to different degrees. According to the IC_50_ values, Cu-ninhydrin complex, [Cu(HL^2^)(H_2_O)_4_].Cl has a better potency impact in comparison with cisplatin which was used as a reference control. This is in harmony with the molecular docking simulation outcomes that predicted a good binding propensity of the Cu-ninhydrin complex with hepatocellular carcinoma protein (*2jrs*). Therefore, the Cu-ninhydrin complex should be deemed as a potential chemotherapeutic agent for hepatocellular cancer.

**Graphical Abstract:**

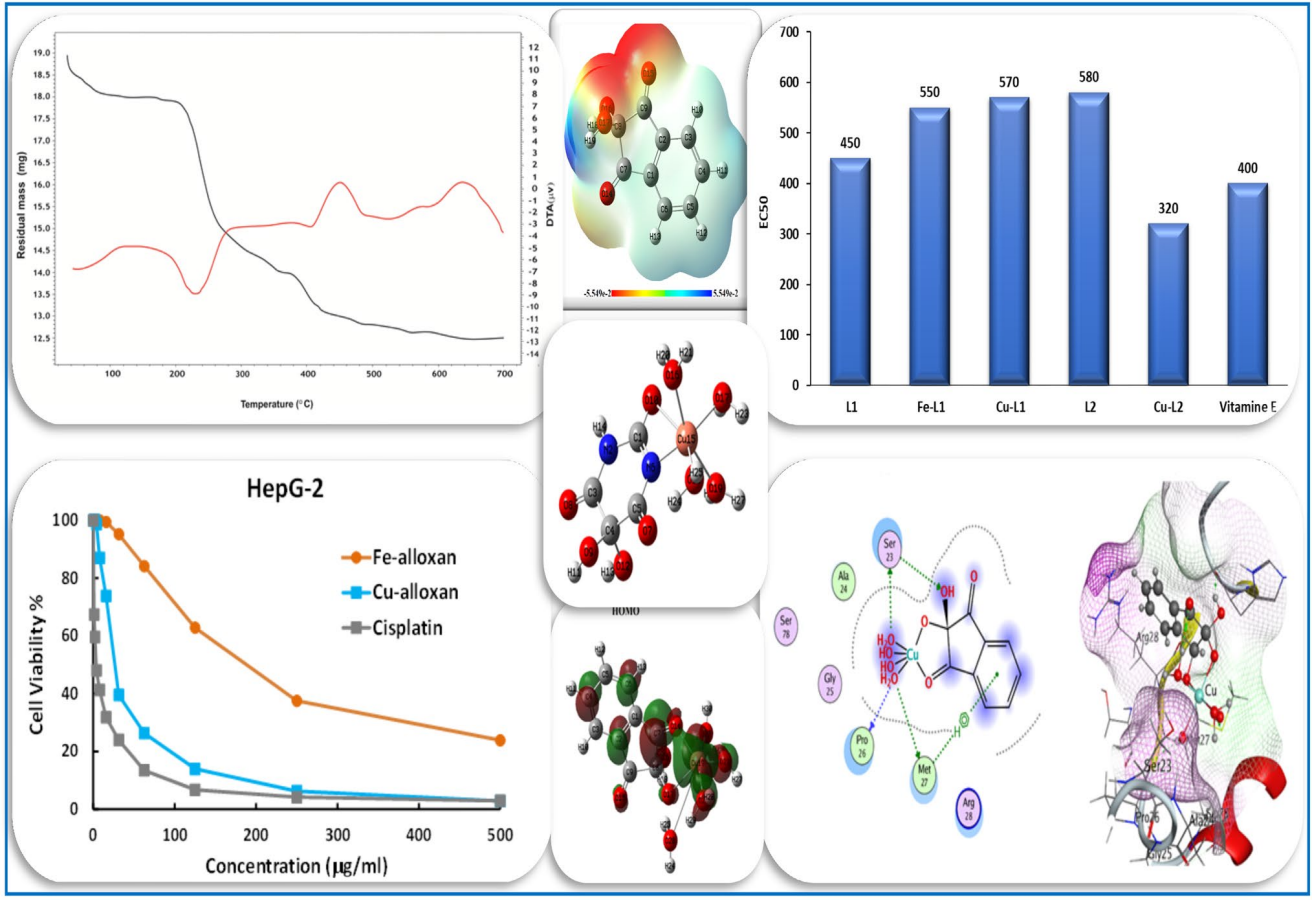

**Supplementary Information:**

The online version contains supplementary material available at 10.1007/s10904-023-02661-5.

## Introduction

Alloxan and ninhydrin are privileged compounds because of their inclusive range of applications in biological and organic synthesis fields [1–5]. Alloxan is considered an example of a pyrimidine derivative while ninhydrin possesses an indanone core moiety that is found in many natural products. Detailed investigations of these compounds are essentially important to correlate the relationship between their structures and their biological efficacy [6, 7]. As well, pyrimidine derivatives are known for many diverse applications as antioxidants, anti-inflammatory, and antiviral drugs [8, 9]. Especially with the current coronavirus threat, many research efforts are converging to find more effective antiviral agents by making different changes in the pyrimidine building block [9]. In our group, a series of papers have been reported to throw light on the chemistry of the biologically active pyrimidine derivatives and their metal complexes [10–12]. Also, many efforts have been done to apply ninhydrin in fluorescence techniques by developing new fluorogenic systems such as the human serum albumin assay [13]. Some ninhydrin-based fluorophores were also studied to behave as selective ligands for certain metal ions [14].

For decades, there have been a growing number of research studies on the complexation of alloxan since it possesses more than one equivalent chelation site as a good example of alterdentate ligands [15–17]. Possible metal ion exchange between these sites occurs *via* intra- or intermolecular pathways [18]. Nevertheless, the involvement of ninhydrin in the isolation of mononuclear metal complexes is rare in the literature [19, 20]. Besides, there is increasing attention to designing and developing novel non-platinum metal complexes to overcome the unsolved clinical problems combined with platinum-based chemical drugs [21, 22]. In continuation, the main objective of this research aims to prepare a series of metal complexes derived from alloxan or ninhydrin for diverse applications in the hope of innovating low-cost anti-pathogenic or anticancer candidates and overcoming the problems associated with the use of existing therapeutics.

## Experimental

### Materials, Instruments, and Procedures

Alloxan and ninhydrin ligands were supplied by Memphis and Squibb companies. The metal salts used in the synthesis of complexes: Fe_2_(SO_4_)_3_.3H_2_O, CoSO_4_.7H_2_O, NiSO_4_.6H_2_O, CuCl_2_.2H_2_O, ZrCl_4_, and (NH_4_)_6_Mo_7_O_24_.4H_2_O are supplied from Sigma-Aldrich. Mammalian hepatocellular cancer cell line (HepG-2) was obtained from VACSERA Tissue Culture Unit (Egypt). All other chemical materials and solvents are of analytical reagent grade. All solutions used throughout the experiments were prepared freshly in ultra-pure water obtained from deionized water.

The infrared spectra were recorded using a potassium bromide disc on Bruker tensor 37 FT-IR spectrophotometer covering the frequency range of 400–4000 cm^–1^. The electronic spectra of the solid complexes were measured using the nujol mull technique on a double beam UV-Visible spectrophotometry (T70-UV/Vis) PG instrument covering the wavelength range 190–900 nm. Molar magnetic susceptibilities, corrected for diamagnetism using Pascal^’^s constants, were determined at room temperature (298 °K) using Faraday^’^s method. Hg[Co(SCN)_4_] was used for calibrating the Gouy tubes [23]. Differential thermal analysis (DTA), thermogravimetric analysis (TGA), and differential scanning calorimetry (DSC) were carried out at a heating rate of 10 °C min^–1^ using Bruker LINSEIS STA PT 1000 under N_2_ flow of 20 cm^3^ min^–1^. The instrument is located at the central laboratory, Faculty of Science, Alexandria University, Alexandria, Egypt.

The metal content was determined by a convenient complexometric titration procedure with standard EDTA [24], and by atomic absorption technique at the central lab, Alexandria University. Elemental analyses (CHN) were performed at the microanalytical unit, Faculty of Science, Cairo University on a Perkin Elmer 2400 elemental analyzer. The analysis of chloride ions was performed by the familiar Volhard method using a standard AgNO_3_ solution and ferric alum as an indicator [24]. The content of sulfate was determined by adding barium chloride solution which results in BaSO_4_ precipitation in a turbidimetric form of uniform size. This process is improved in presence of glycerol, NaCl, and HCl acid. The absorbance of the resulting BaSO_4_ is quantified from a standard calibration curve based on spectrophotometer readings at 420 nm of different previously prepared standard concentrations [25]. The analytical data and physical properties of the synthesized complexes are represented in Table 1.

**Table 1:** Color and elemental analyses of alloxan monohydrate (H_2_L^1^) and ninhydrin (H_2_L^2^) complexes

Complex	Formula Color	% Calculated / (Found)					
		M	C	H	N	Cl	SO_4_
Fe-alloxan [Fe(HL^1^)(H_2_O)_4_].SO_4_	C_4_H_11_N_2_O_13_SFe Dark yellow	14.58 (15.07)	12.54 (12.88)	2.89 (2.54)	7.31 (7.67)	–	25.08 (25.57)
Co-alloxan [Co(H_2_L^1^)(H_2_O)_4_].SO_4_	C_4_H_12_N_2_O_13_SCo Buff	15.22 (15.29)	12.41 (12.38)	3.12 (3.09)	7.24 (7.19)	–	24.80 (24.90)
Ni-alloxan [Ni(H_2_L^1^)(H_2_O)_2_].SO_4_	C_4_H_8_N_2_O_11_SNi Pale green	16.73 16.57)	13.69 (13.67)	2.30 (2.28)	7.98 (8.11)	–	27.42 (27.38)
Cu-alloxan [Cu(HL^1^)(H_2_O)_4_].Cl	C_4_H_11_ClN_2_O_9_Cu Greenish brown	19.25 (18.92)	14.55 (14.05)	3.36 (2.97)	8.49 (8.48)	10.74 (10.62)	–
Zr-alloxan [Zr(HL^1^)Cl_3_(H_2_O)]	C_4_H_5_Cl_3_N_2_O_6_Zr Dark yellow	24.35 (23.99)	12.82 (12.47)	1.35 (1.74)	7.48 (7.03)	28.39 (27.93)	–
Mo-alloxan [MoO_2_(HL^1^)(H_2_O)OH]	C_4_H_6_N_2_O_9_Mo Dark blue	29.79 (29.29)	14.92 (14.64)	1.88 (1.68)	8.70 (8.47)	–	–
Fe-ninhydrin [Fe(HL^2^)(H_2_O)_4_].SO_4_	C_9_H_13_O_12_SFe Pale brown	13.92 (14.36)	26.95 (27.13)	3.27 (3.08)	–	–	23.95 (24.11)
Co-ninhydrin [Co(H_2_L^2^)(H_2_O)_4_].SO_4_	C_9_H_14_O_12_SCo Pale orange	14.54 (14.15)	26.68 (26.32)	3.48 (3.88)	–	–	23.70 (23.24)
Ni-ninhydrin [Ni(H_2_L^2^)(H_2_O)_2_].SO_4_	C_9_H_10_O_10_SNi Pale blue	15.91 (15.55)	29.30 (28.91)	2.73 (3.12)	–	–	26.08 (25.87)
Cu-ninhydrin [Cu(HL^2^)(H_2_O)_4_].Cl	C_9_H_13_ClO_8_Cu Pale brown	18.25 (17.91)	31.05 (30.07)	3.76 (3.32)	–	10.18 (10.43)	–
Zr-ninhydrin [Zr(HL^2^)Cl_3_(H_2_O)]	C_9_H_7_Cl_3_O_5_Zr Dark yellow	23.23 (22.78)	27.52 (27.05)	1.80 (1.35)	–	27.08 (26.71)	–
Mo-ninhydrin [MoO_2_(HL^2^)(H_2_O)OH]	C_9_H_8_O_8_Mo Brown	28.21 (28.40)	31.78 (31.73)	2.37 (2.49)	–	–	–

H_2_L^1^: Alloxan monohydrate (C_4_H_4_N_2_O_5_); H_2_L^2^: Ninhydrin (C_9_H_6_O_4_)

GAUSSIAN 09 software was used for the calculations of molecular orbital parameters based on the DFT(B3LYP/6-31G) level of theory. The optimized structures were visualized in GAUSSIAN-VIEW. Docking investigation was attained by MOE 2015.10 software. The initial steps to prepare the tested compound for the docking process included hydrogen atoms addition, removal of water molecules, atomic charges clarifying, and then energy minimization by MMFF94x force field [26].

### Biological Activity Studies

#### Antimicrobial Activity

Screening tests regarding the in vitro inhibition zone measurement in mm were carried out by the well diffusion method [27]. Briefly, the inoculum suspension was prepared from colonies grown overnight on an agar plate in 10 ml of Mueller-Hinton agar medium (Merck, Germany). A sterile swab was immersed in the bacterial suspension and used to inoculate agar plates. Each tested compound was dissolved in dimethyl sulfoxide (DMSO, 5 mg/ml). 100 µl was tested and each inhibition zone was measured around each well after 24 h at 37 °C. Controls using DMSO were done, and tests were duplicated by using ketoconazole and gentamycin as references for antifungal and antibacterial activities, respectively.

#### Antioxidant Activity

The stable radical 2,2′-diphenyl-1-picrylhydrazyl (DPPH) was used as a reagent for spectrophotometric assay. 200 µl serial concentrations of each sample (150, 300, 450, 600, 750, 900, 1000 mg) were mixed with 1 ml of DPPH (0.0025 g/ml in methanol), each separately. The mixture was shaken vigorously and then kept in dark for half an hour, the decrease in absorbance of each mixture was measured spectrophotometrically at 517 nm. Blank was prepared in DPPH without antioxidants, while vitamin E was used as a synthetic reference [28]. The effective concentration of a sample that needed to scavenge DPPH radical by 50% (EC_50_ value) was obtained by linear regression analysis of dose-response curve plotting between % scavenging and concentrations. Antioxidant tests were performed at the biochemistry laboratory, Faculty of Science, Alexandria University, Alexandria, Egypt.

#### Anticancer Activity

Four of the synthesized metal complexes were evaluated as a preliminary study for their human tumor cell growth inhibitory activity against hepatocellular carcinoma cells (HepG-2). Cytotoxicity evaluation using crystal violet viability assay was accomplished as described [29]. Cisplatin was utilized as positive control under the same measurement conditions. Antimicrobial and cytotoxicity evaluations were carried out by Regional Center for Mycology & Biotechnology (RCMP) at Al-Azhar University, Cairo, Egypt.

### General Procedure for the Synthesis of Metal Complexes

All complexes were prepared in a similar procedure. The required weight of the transition metal chloride or sulfate salt (2 mmole) was dissolved in 10 ml distilled water and mixed with the required weight of ligand (2 mmole) dissolved in 25 ml methanol. Then, drops of ammonia solution (1:1) were added to make the medium slightly basic (pH 8.0) except in the case of iron complexes. The reaction mixture was heated to 60 °C for 1 h with continuous stirring, then cooled where a precipitate was formed, filtered then dried in an oven at 90 °C.

## Results and Discussion

### Characterization of the Synthesized Metal Complexes

Characterization of the investigated compounds was elucidated by different microanalytical, spectroscopic tools, magnetic susceptibility, and thermal studies.

#### FT-IR Spectra of Ligands and Their Complexes

**Fig. 1:**
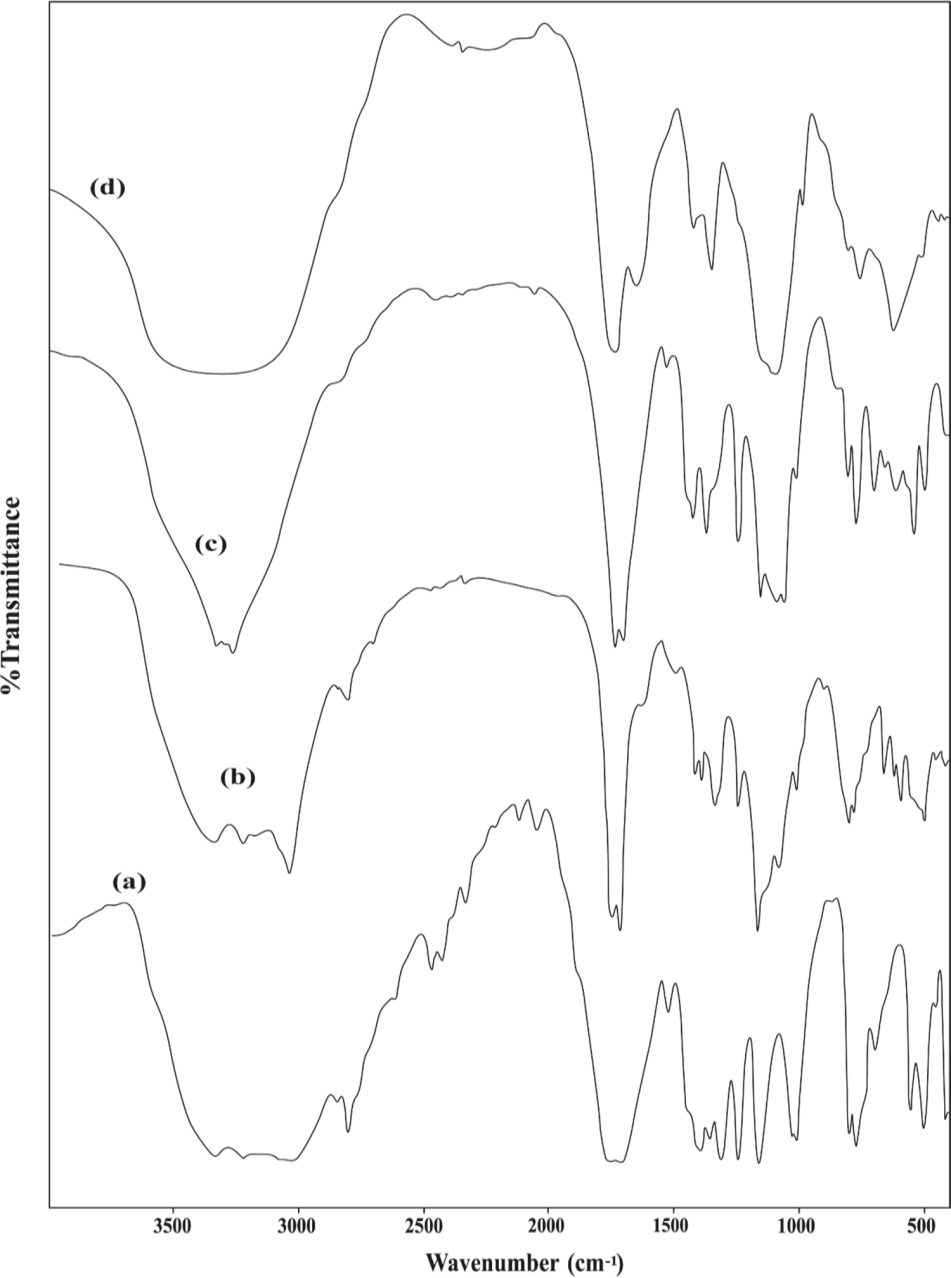
FT-IR of **a** alloxan, and its **b** Fe, **c** Co, **d** Ni complexes

**Fig. 2:**
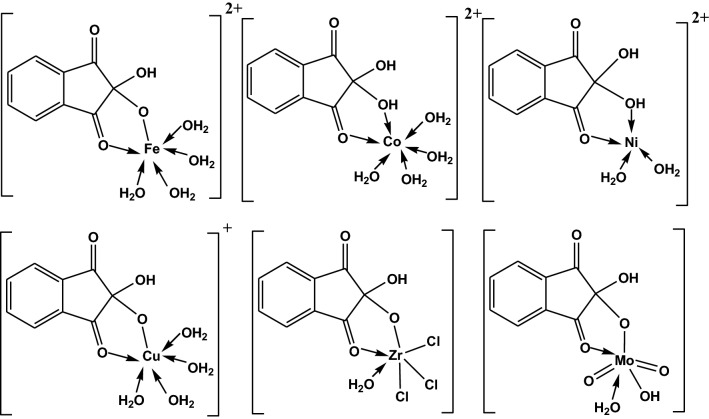
Suggested structures of inner sphere metal ninhydrin complexes

**Fig. 3:**
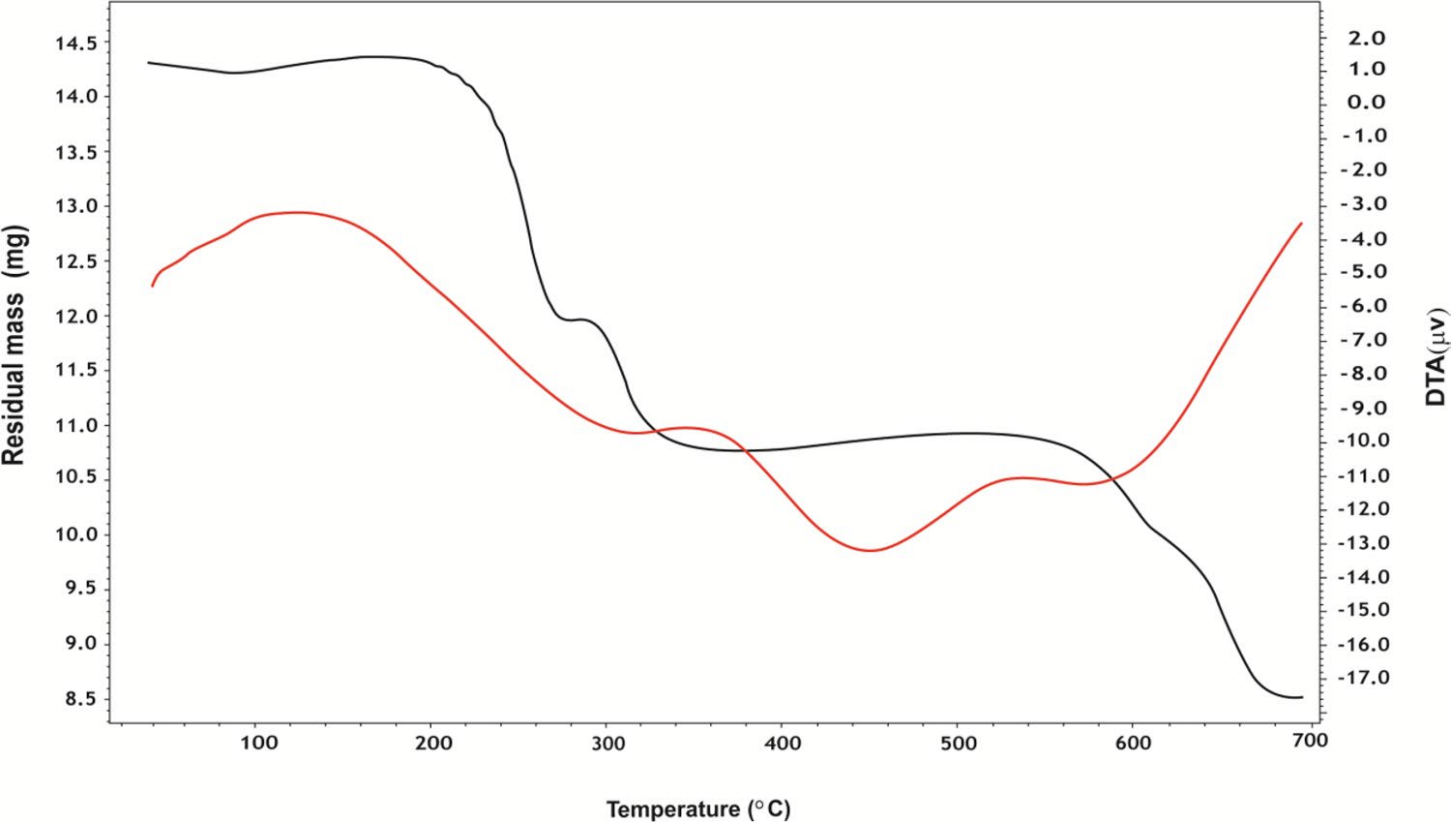
TGA and DTA curves of Fe-alloxan complex

Detailed interpretations of infrared spectral data with some important characteristic assignments are elaborated, Figs. 1, 1S, 2S and 3S and Tables 1S, 2S in the supplementary electronic information. The infrared spectra of the prepared complexes were compared with that of the free ligands to shed light on the bonding mode of the central metal ion with the surrounding ligand molecules.

The IR spectra of alloxan and its metal complexes exhibit strong broad bands in the range of 3448 − 3041 cm^–1^, Figs. 1 and 1S, corresponding to overlapping between ν(OH) and ν(NH) vibrations [15]. The broad feature of the bands in this region could be taken as evidence of the coordination with H_2_O molecules and the presence of intramolecular hydrogen bonds [17]. This is convenient with the analytical data and TGA results where all complexes possess some inner sphere water molecules. Also, intense carbonyl bands appeared in the alloxan IR spectrum with maxima at 1767 cm^–1^ ν(C(2) = O) and 1715 cm^–1^ with a shoulder at 1685 cm^–1^ [overlapped ν(C(4) = O) & ν(C(6) = O)]. These bands suffer either disappearance, position shift, or lower intensity in the case of IR spectra of complexes, Table 1S. Meanwhile, the appearance of the ν(N = C–O) band at the range of 1639–1625 cm^–1^ in all complexes -except Co & Ni- could be assigned to the displacement of the proton between the N(1)H and C(2) = O with enol formation before N(1) & O(2) chelation. This does not exclude the possibility of chelation of metal ions with N(3) & O(4) with similar H-displacement as alloxan is an alterdentate ligand as mentioned before [18]. Besides, the appearance of new low-frequency bands at 636–595 and 501–481 cm^–1^ in the spectra of metal-alloxan complexes can be assigned to ν(M–O) and ν(M–N) sustaining the complex formation [30].

The fundamental vibration bands of ninhydrin and its metal complexes are shown in Table 2S and Figs. 2S and 3S. The aromatic ν(C–H) stretching appears at wavenumber 3089 cm^–1^ while δ(C–H) bending modes are observed at 1255, 1185 and 1065 cm^–1^ (in-plane), and 740 cm^–1^ (out of plane) [31]. The broad band at 3300 cm^–1^ in the spectra of free ninhydrin due to ν(OH) is shifted in the spectra of metal-ninhydrin complexes suggesting coordination through the oxygen atom of one of the hydroxyl groups in the prepared complexes. Also, the infrared spectrum of ninhydrin exhibits the ν(C = O) at 1749 cm^–1^ which suffers an obvious lowering in intensity in the spectra of its metal complexes. This negative shift indicates the involvement of one of the two carbonyl groups of ninhydrin in coordination with the metal ion.

Likewise, the appearance of the new band at 520–472 cm^–1^ in the spectra of all metal-ninhydrin complexes, which is absent in the spectra of free ninhydrin ligand, can be assigned to ν(M–O) [30]. This reveals that ninhydrin can react with metal ions as a bidentate ligand involving hydroxyl oxygen and carbonyl oxygen either C(1) = O or C(3) = O since ninhydrin is an alterdentate ligand as alloxan [18].

Notably, Mo-alloxan and Mo-ninhydrin complexes, Figs.  1S and 3S, display a strong band at 937 and 935 cm^–1^, respectively, that is designated to ν(Mo = O) confirming the presence of such bond in their structures, Table 1 [32].

#### **Electronic Spectra, Magnetism, and Conductivity Measurements**

**Fig. 4:**
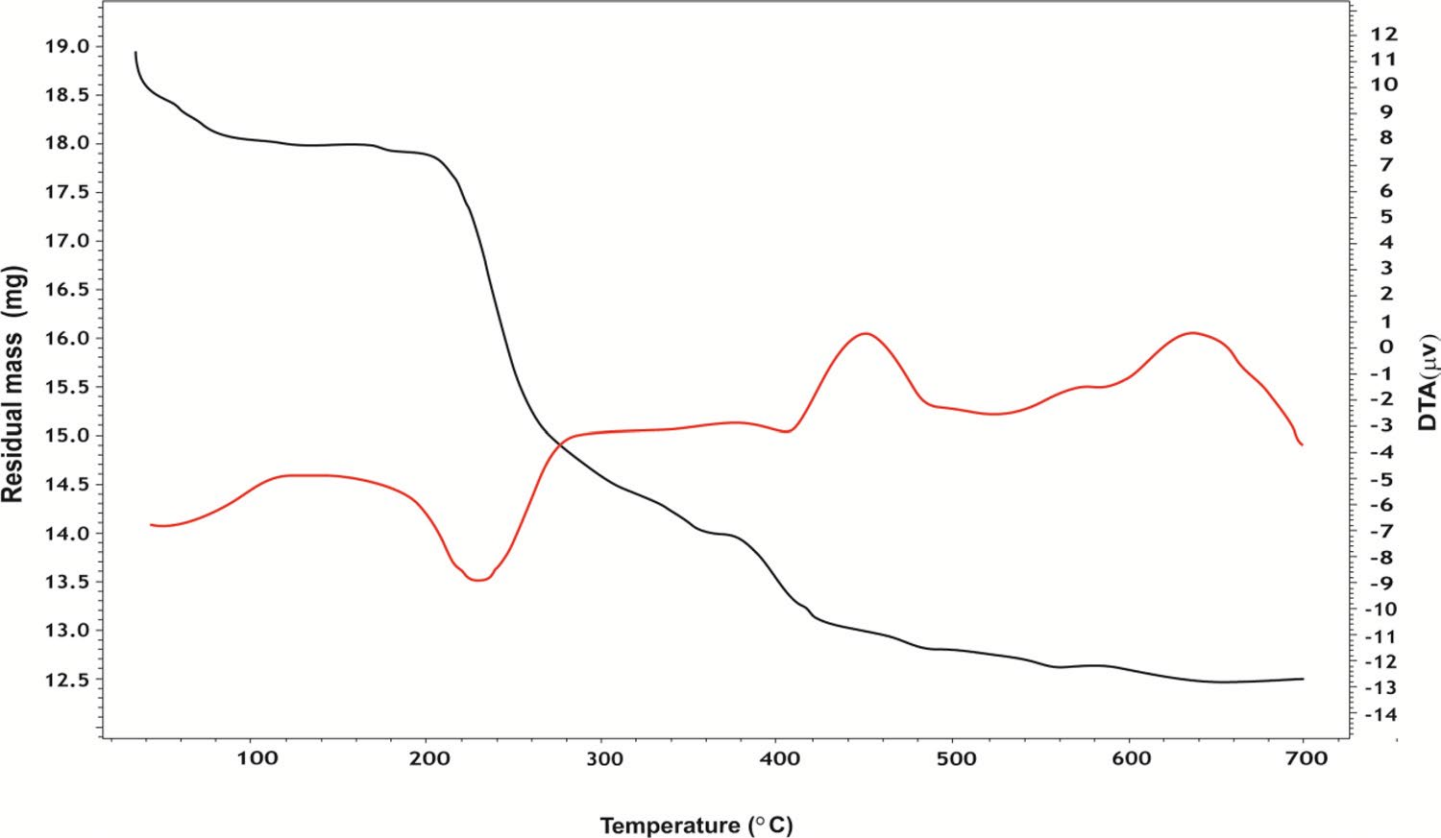
TGA and DTA curves of Mo-ninhydrin complex

**Fig. 5:**
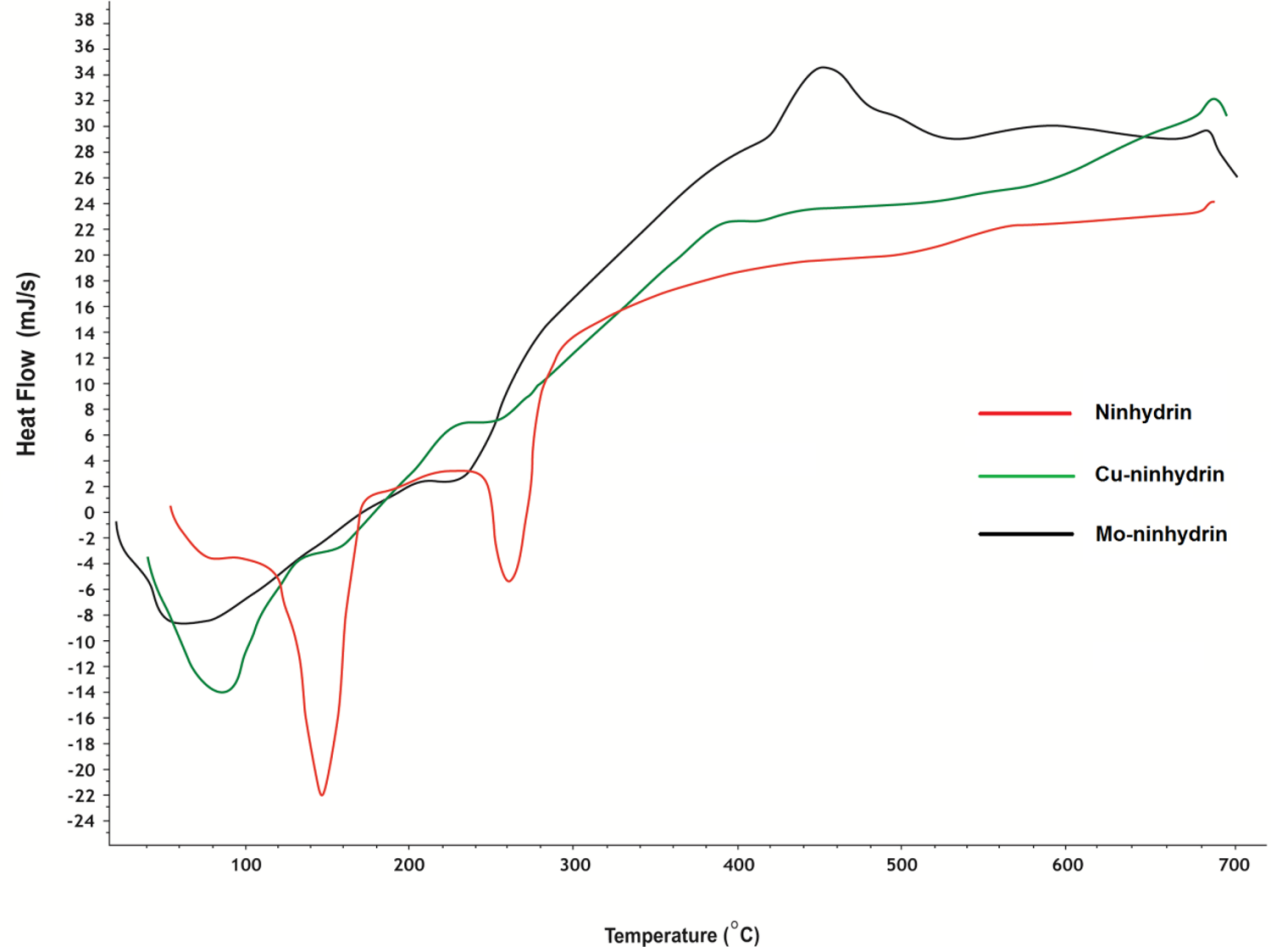
DSC curves of ninhydrin and its Cu and Mo complexes

**Fig. 6:**
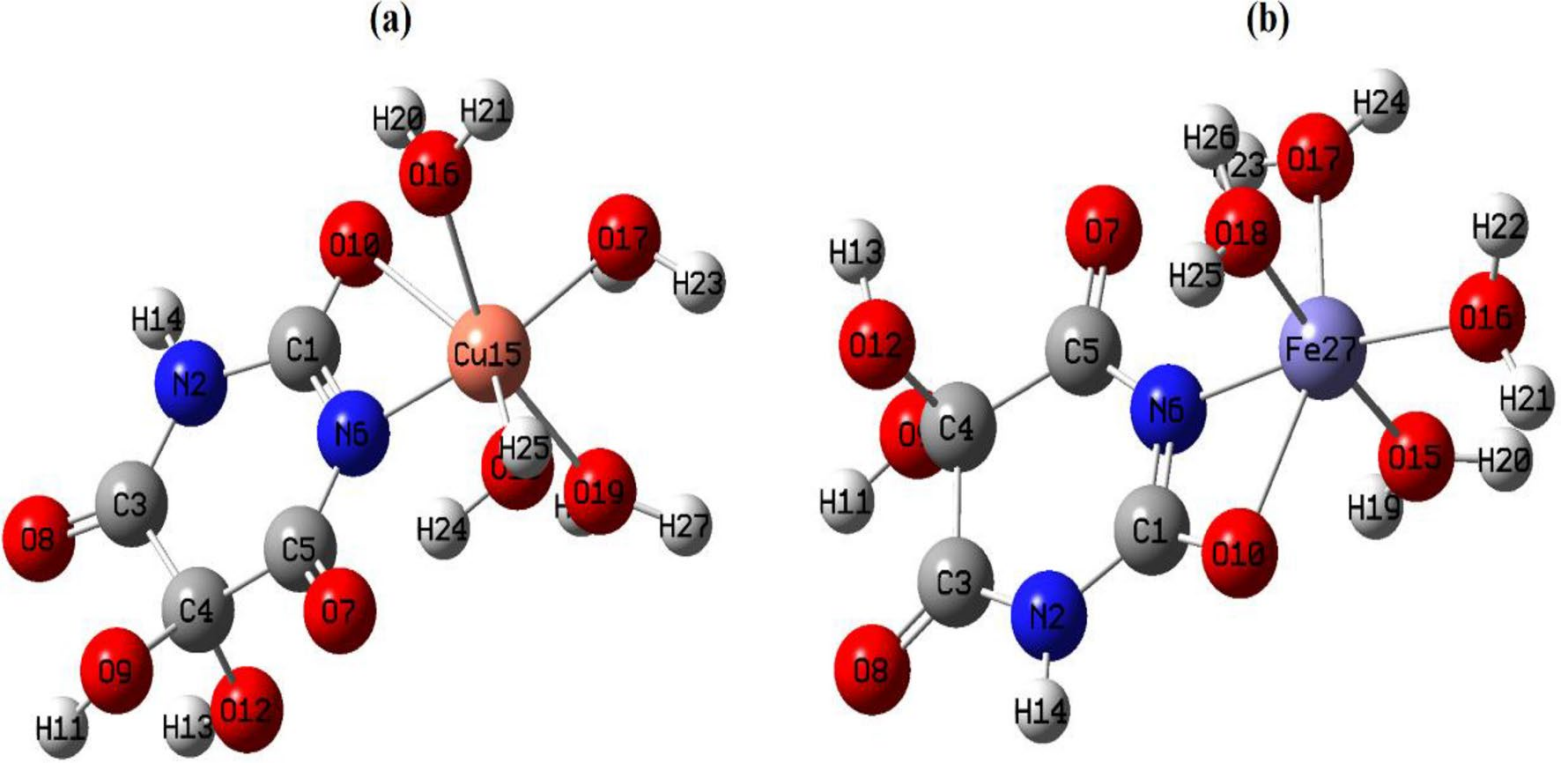
Optimized structure of **a** Cu-alloxan and **b** Fe-alloxan

The UV-Vis spectra of the ligands (H_2_L^1^ and H_2_L^2^) and their metal complexes are collected in Table 2; Figs. 4S and 5S. The electronic absorption spectra of the iron complexes of the entitled ligands showed two bands at 270–275  and 345–350 nm due to π → π* and CT (L→M), respectively. The room temperature *µ*_eff_ values of 2.21–2.22 B.M typified the existence of octahedral configuration in a low spin state [33]. The structure of these iron (III) complexes is constructed depending on the bidentate nature of alloxan or ninhydrin ligands with the existence of four water molecules in the inner sphere, and one SO_4_^2−^ group in the outer sphere, Figs. 2 and 6S.

The electronic spectra of cobalt complexes showed three bands. The first two bands in the ranges 260–280 and 300–320 nm could be assigned to π → π* and LMCT in that order. Nevertheless, the third weak intense band at 380–386 nm is due to the ^4^T_1g_(F) → ^4^T_1g_(P) transition typifying octahedral geometry. The room temperature *µ*_eff_ value of 5.02–5.03 B.M implied a high spin environment for the two complexes with weak coordination bonds [33]. However, the electronic absorption spectra of the Ni-alloxan complex showed two bands at 290 and 480 nm due to π → π* and ^3^T_1_ → ^3^T_1_(P) transitions. The room temperature *µ*_eff_ values of 4.11–4.12 B.M, Table 2, typified the presence of the two nickel complexes derived from alloxan and ninhydrin in perfect tetrahedral geometry [34].

The electronic absorption spectra of the two copper complexes showed three bands in the range of 260 to 385 nm. The broadening of the third CT band expands to 460 nm inside the visible region. The broad feature of this band makes it difficult to distinguish any weak d-d transitions in this region [22]. The observed values of *µ*_eff_ for copper (II) complexes are 2.30–2.31 B.M which is higher than the spin-only value corresponding to one unpaired electron 1.73 B.M. This could be attributed to the orbital contribution to the spin of octahedral complexes [33]. The Oh structure is established by the bidentate mode of the ligands and four water molecules in the inner sphere with the presence of one chloride ion in the outer sphere, Figs. 2 and 6S.

As for all d^0^ configurations, no characteristic d-d transitions are expected for Zr^4+^ and Mo^6+^ ions in visible light > 380 nm. Any found bands at lower wavelengths (in the UV region) could be assigned to CT transitions of the type L → M, or π→π^*^and n → π^*^ transitions of the organic part of the complexes [35]. The diamagnetic feature of zirconium (IV) and molybdenum (VI) complexes proposed that these ions exhibit octahedral coordination as similarly reported complexes [32, 35].

The molar conductance (*Λ*_m_) of the isolated complexes was determined. The measurement was done in ethanol of 10^–3^ M solution at 25 °C. The *Λ*_m_ values for Mo and Zr complexes are comparatively low (9.7–19.8 Ω^–1^ cm^2^ mol^–1^ in the case of alloxan complexes), signifying the non-electrolytic manner of these complexes, Table 2 [36]. However, the molar conductance is of relatively higher values for Fe, Co, Ni, and Cu complexes (49.5–207 Ω^–1^ cm^2^ mol^–1^ in the case of ninhydrin complexes), supporting the electrolytic property of these complexes and the existence of SO_4_^2−^ and Cl^−^ ions in the outer sphere, Figs. 2 and 6S [37]. It is worth mentioning that different complexes of alloxan (H_2_L^1^) have been isolated in previous studies [16, 17]. For instance, alloxan was bound to Fe(III) as a dibasic bidentate ligand [17], [Fe(L^1^)(H_2_O)_3_Cl].2H_2_O, and not in the monobasic bidentate fashion, [Fe(HL^1^)(H_2_O)_4_].SO_4_, as in our case. This could be attributed to the variation in the pH of the medium during the synthesis process and the type of counter ions (Cl^−^ or SO_4_^2−^) of the used metal salt [22, 38].

**Table 2:** Room temperature (298˚C) effective magnetic moment values, λ_max_ (nm), and molar conductance data of the synthesized metal complexes

Complex	λ_max_ (nm)	*μ*_eff_ B.M	Geometry	*Λ*_m_ Ω^–1^ cm^2^ mol^–1^
[Fe(HL^1^)(H_2_O)_4_].SO_4_	275, 345	2.22	Oh	59.0
[Co(H_2_L^1^)(H_2_O)_4_].SO_4_	280, 320, 385	5.03	Oh	69.3
[Ni(H_2_L^1^)(H_2_O)_2_].SO_4_	290, 300, 480	4.12	Td	49.5
[Cu(HL^1^)(H_2_O)_4_].Cl	270, 380, 410	2.31	Oh	89.1
[Zr(HL^1^)Cl_3_(H_2_O)]	270, 320	Dia.	Oh	19.8
[MoO_2_(HL^1^)(H_2_O)OH]	260, 310	Dia.	Oh	9.7
[Fe(HL^2^)(H_2_O)_4_].SO_4_	270, 350	2.21	Oh	170
[Co(H_2_L^2^)(H_2_O)_4_].SO_4_	260, 300, 380	5.02	Oh	49.5
[Ni(H_2_L^2^)(H_2_O)_2_].SO_4_	250, 295	4.11	Td	108
[Cu(HL^2^)(H_2_O)_4_].Cl	260, 320, 380	2.30	Oh	207
[Zr(HL^2^)Cl_3_(H_2_O)]	275, 320	Dia.	Oh	31.6
[MoO_2_(HL^2^)(H_2_O)OH]	265, 320, 380	Dia.	Oh	29.2

#### Thermal Studies

**Fig. 7:**
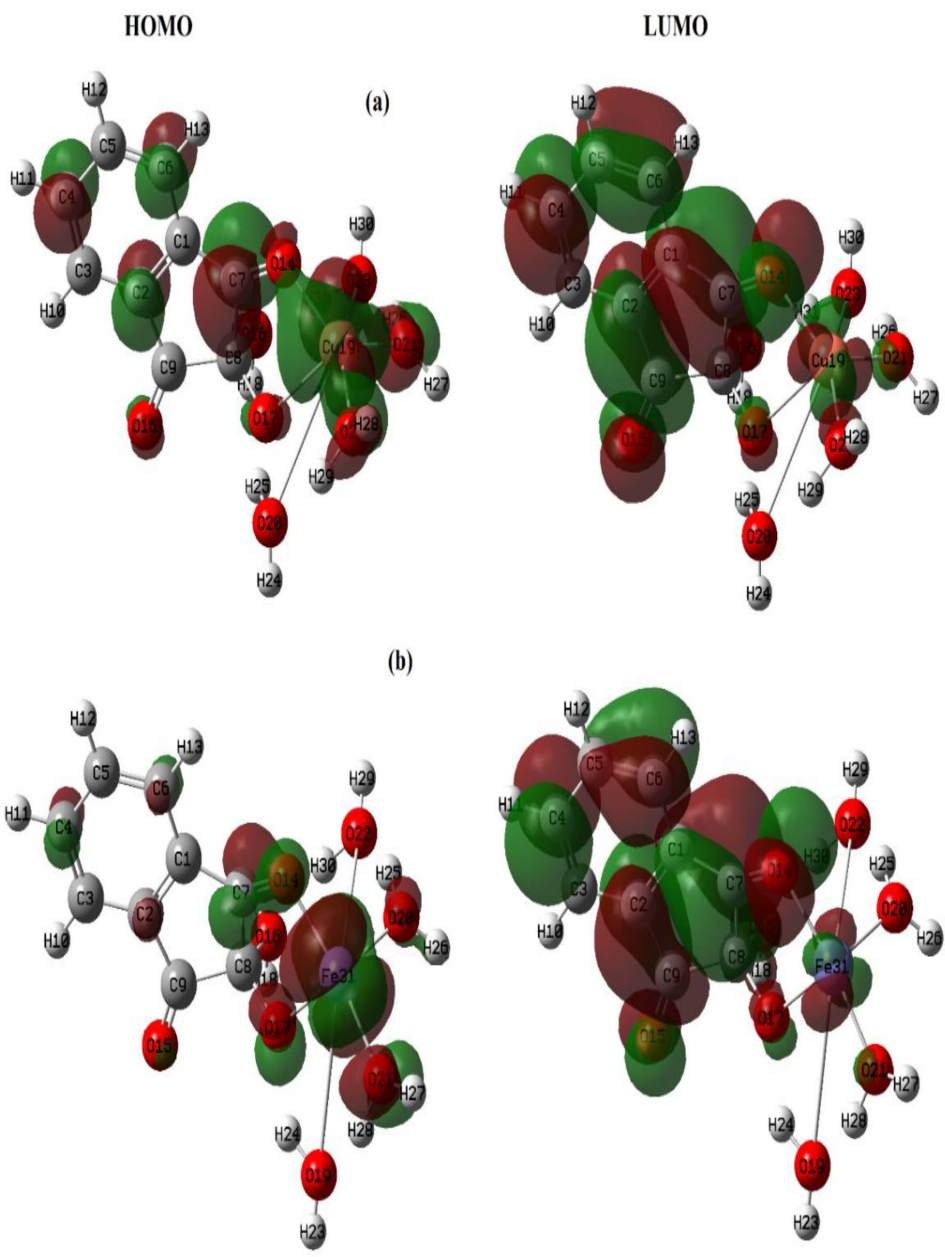
LUMO and HOMO **a** Cu-ninhydrin and **b** Fe-ninhydrin

**Fig. 8:**
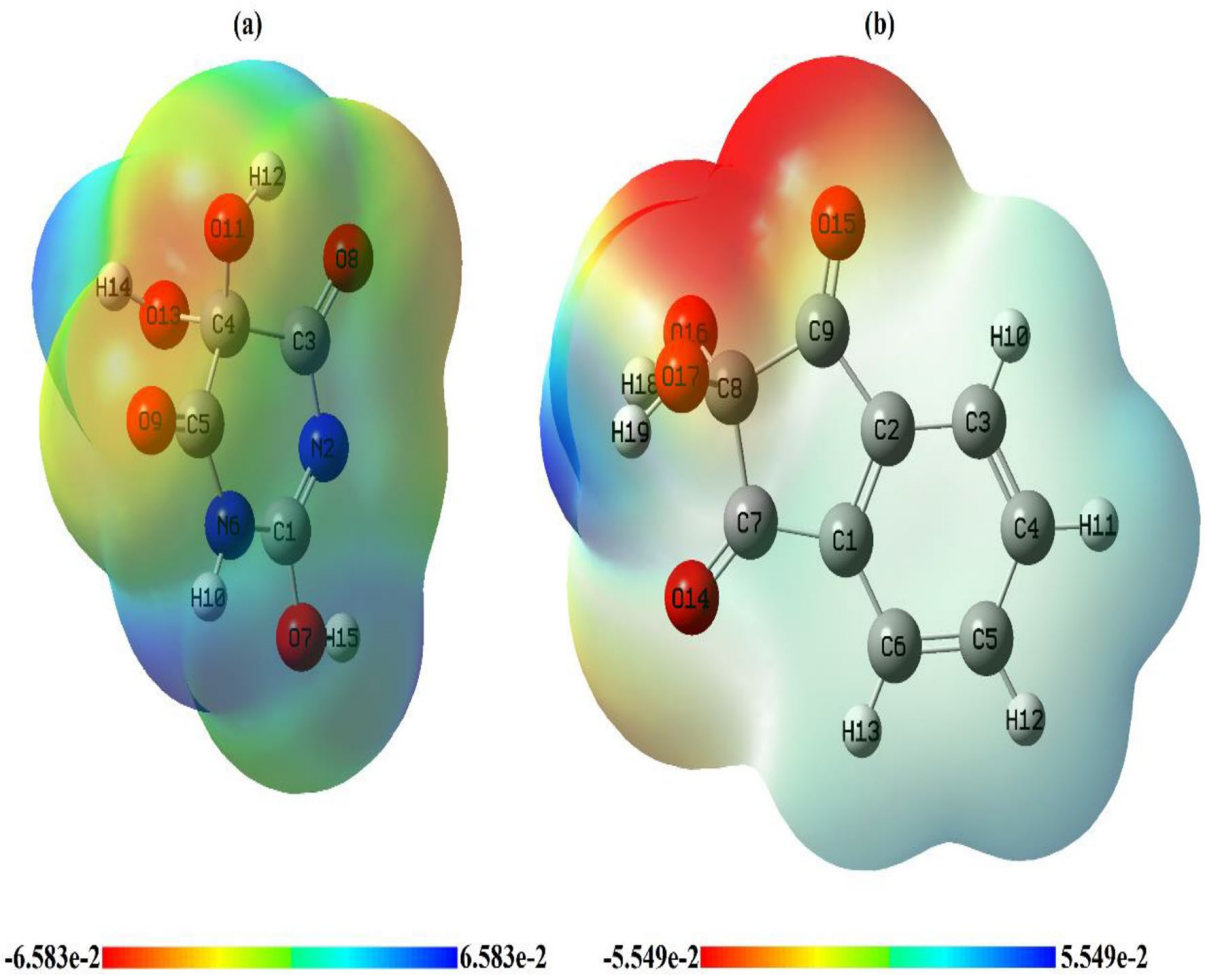
Molecular electrostatic potential maps of **a** alloxan and **b** ninhydrin

**Fig. 9:**
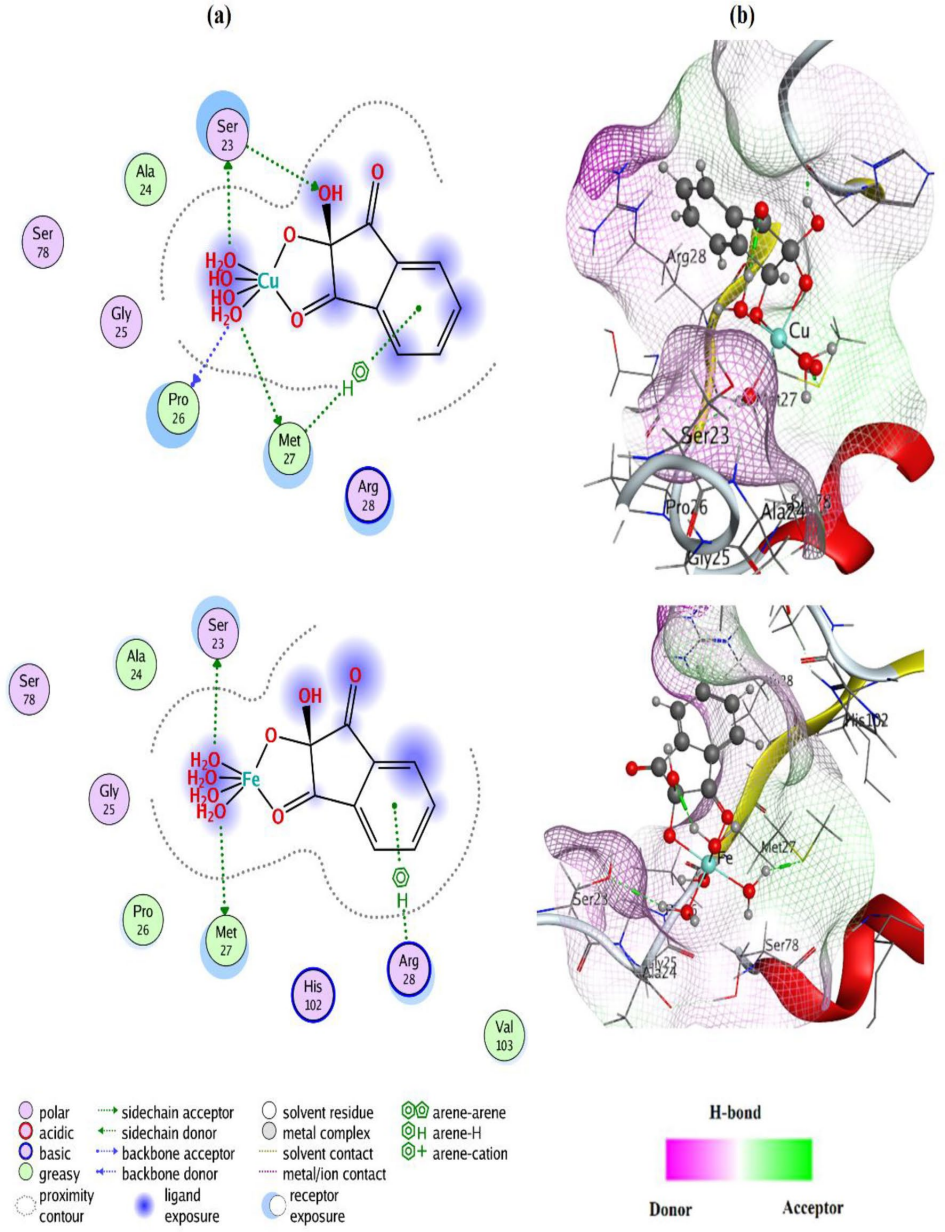
Ligand-receptor interactions **a** and receptor lipophilicity maps **b** of the best-docked poses of Cu-ninhydrin and Fe-ninhydrin complexes

**Fig. 10:**
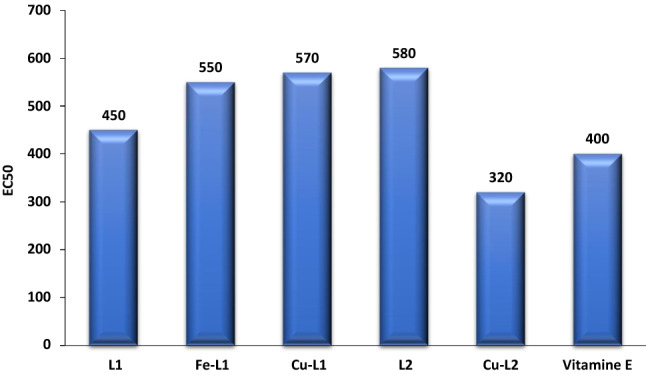
EC_50_ values for alloxan, ninhydrin, and some complexes compared to Vitamin E as standard; Sample code: L^1^: Alloxan, Fe-L^1^: Fe-alloxan, Cu-L^1^: Cu-alloxan, L^2^: Ninhydrin, Cu-L^2^: Cu-ninhydrin

**Fig. 11:**
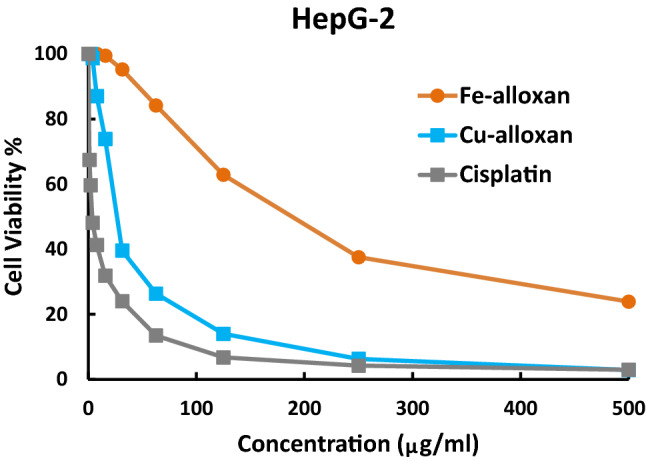
The inhibitory dose curves of Fe-alloxan and Cu-alloxan compared to *cis*-platin against HepG-2 cell line

TGA, DTA, and DSC studies of alloxan, ninhydrin, and some of their complexes, Figs. 3, 4, 5 and 7S–11S, have been carried out to confirm the proposed structures and the number of water molecules present in the inner and/or outer spheres of the complexes as well as to know their general decomposition patterns and to evaluate the thermodynamic parameters (∆H*, ∆S*, and ∆G*) using Eyring equations and Coats-Redfern method [39], Table 3S, associated with each degradation step, Table 3.

In general, the TGA curve of the complexes displayed a three-stage decomposition pattern except for the [Zr(HL^1^)Cl_3_(H_2_O)] complex which exhibited a two-stage decomposition process. As a demonstrating example, the thermogram of [Fe(HL^1^)(H_2_O)_4_].SO_4_ complex showed three distinct decomposition stages; the first stage occurs in the temperature range (37–286 °C) with weight loss (18.6%) due to dehydration of four coordinated water molecules (Calc. 18.8%). The second stage occurs in the temperature range (286–534 °C) with weight loss (25.4%) giving out the outer sphere sulfate moiety (H_2_O and SO_3_, Calc. 25.6%). Finally, the third stage occurs at (534–697 °C) with weight loss (37.2%) to remove the rest of the organic ligand molecule (C_4_HN_2_O_4_) leaving FeO residue, Table 3. The small discrepancy between the calculated and found weight losses in the second and third stages is taken as an indication of overlapping of the decomposition steps in these stages. It is worth stating that the formation of these residues at such a high temperature range (700 °C) could be taken as an indication of the higher thermal stability of the studied complexes.

The thermal decomposition of the ninhydrin ligand takes place in two stages of mass loss, Fig.  9S. The first step progresses up to 189 °C which is due to the loss of 2 H_2_O molecules and corresponds to the first endothermic DTA peak at 146.6 °C. Besides, the second endothermic DTA peak at 261.7 °C allocates the ninhydrin melting point [40]. After melting, decomposition continues at higher temperatures until the removal of the rest of the compound as gaseous molecules with almost no residue.

Regarding DTA curves, the first peak of the DTA curve of [Fe(HL^1^)(H_2_O)_4_].SO_4_ complex, Fig. 3, corresponding to the dehydration process of 4 coordinated water molecules takes place in one step in the temperature range (34.6–223.9 °C) with activation energies of 27.6 kJ/mole. However, the DTA curve of the [MoO_2_(HL^2^)(H_2_O)OH] complex, Fig. 4, pointed out that dehydration and loss of CO_2_ processes take place in two successive steps in the temperature range (34.7–279.8 °C), with activation energies of 31.2 and 153.6 kJ/mole, respectively. The other peaks of the DTA curves [MoO_2_(HL^2^)(H_2_O)OH] complex are due to thermal agitation and ligand decomposition stage ending with the formation of MoO_2_ as a final product.

The positive sign of the enthalpy (∆H*) and the free energy (∆G*) of activation in all studied cases, Table  3S, reveals that all decomposition steps are of endothermic and nonspontaneous nature, respectively, where the final residue is of higher energy than that of the started complex. Further, the values of (∆G*) increase considerably for the successive decomposition stages for each compound. This causes an increase in the removal rate of the precedent species than that of the subsequent one [41]. Furthermore, the entropy of activation (∆S*) is of negative value for all decomposition processes implying that the activated complexes are of a more ordered structure compared to the undecomposed complexes [42]. Also, the values of ∆S* are nearly equal, Table  3S, so the reaction was entropy-independent.

DSC is also used to study thermal transitions such as glass transition (T_g_), melting (T_m_), and crystallization (T_g_) temperatures [43], Table 4 S and Figs. 5 and 11 S. The glass transition temperature exhibits a dehydration process of the inner sphere water molecules followed by thermal agitation decomposition. This is in harmony with the TGA findings for these complexes. All the prepared complexes melt above ∼ 250 °C. DSC plot is used to determine the melting temperature through an endothermic transition, where the compounds should absorb heat until all the crystals are melted. T_m_ values of ligands and their complexes are varied between 255 and 450 °C, Table 4 S.

**Table 3:** Thermal decomposition studies of ligands and some of their complexes

Compound	Temp. range (°C)	Peak temp. (°C)	Mass loss (%)		Assignment
			Calc.	Found	
Alloxan C_4_H_4_N_2_O_5_	39–390	184	59.2	58.9	Elimination of H_2_O, C_2_N_2_O_2_
	390–695	505	32.4	32.7	Elimination of (CH_2_O_2_) and formation of C residue
Fe-alloxan [Fe(HL^1^)(H_2_O)_4_].SO_4_	37–286	160	18.8	18.6	Dehydration of 4 inner sphere H_2_O
	286–534	443	25.6	25.4	Elimination of H_2_O, SO_3_
	534–697	604	36.8	37.2	Decomposition of (C_4_HN_2_O_4_) and formation of FeO
Zr-alloxan [Zr(HL^1^)Cl_3_(H_2_O)]	34–362	264	33.5	33.3	Elimination of H_2_O, Cl_2_, HCl
	362–699	454	33.4	33.7	Elimination of (C_4_H_2_N_2_O_3_) and formation of ZrO_2_ as a residue
Ninhydrin C_9_H_6_O_4_	53-189	147	20.2	20.5	Elimination of 2H_2_O molecules
	189–698	443	66.3	66.0	Decomposition of (C_7_H_2_O_2_) and formation of 2C residue
Cu-ninhydrin [Cu(HL^2^)(H_2_O)_4_].Cl	50–190	158	20.7	20.5	Dehydration of 4 inner sphere H_2_O
	190–370	259	10.4	10.3	Elimination of HCl
	370–680	450	46.0	46.4	Elimination of the ligand (C_9_H_4_O_3_) and formation of CuO
Mo-ninhydrin [MoO_2_(HL^2^)(H_2_O)OH]	35–145	90	10.6	10.7	Dehydration of 2H_2_O
	145–297	233	12.9	13.0	Elimination of CO_2_
	297–697	449	38.8	38.7	Elimination of (C_8_H_4_O_2_) and formation of MoO_2_

### Molecular Modeling Studies

#### Structural Optimization Using DFT

**Fig. 12:**
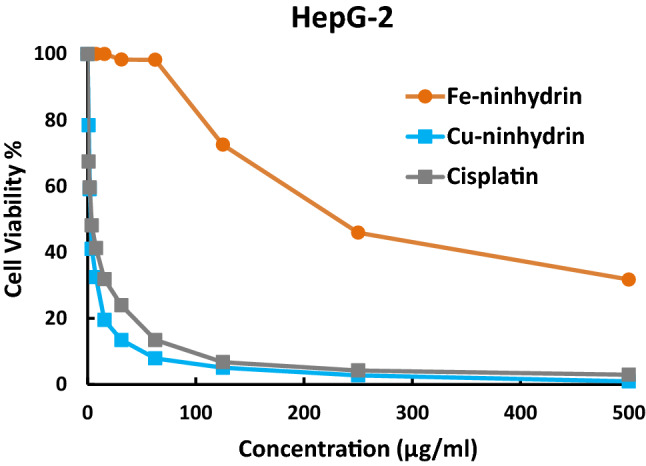
The inhibitory dose curves of Fe-ninhydrin and Cu-ninhydrin compared to cisplatin against HepG-2 cell line

Density functional theory (DFT) was employed to calculate the molecular parameters of the best optimized conformations of the studied ligands (H_2_L^1^ & H_2_L^2^) and some of their metal complexes. DFT is considered a reliable method to identify the nucleophilic and electrophilic sites as well as the molecular polarity that affects their reactivity with metal cations or biological receptors. The structure of the ligands and their Cu(II) and Fe(III) complexes as illustrative examples were optimized to the lowest energy conformers with DFT-B3LYP/ 6-31G basis set using Gaussian 09 program [44, 45]. The theoretical data of the investigated compounds are collected in Table 4; Figs. 6, 7, 8, 9 and 12S–17S.

**Table 4:** The molecular parameters of the ligands and their Cu(II) and Fe(III) complexes

Compounds	Total Energy (Hartree)	Dipole moment (Debye)	E_HOMO_ (eV)	E_LUMO_ (eV)	ΔE (eV)	η (eV)	S (eV^− 1^)	µ (eV)	χ (eV)	ω (eV)
Alloxan (H_2_L)	−640	3.66	−7.685	−2.681	5.004	2.502	0.199	−5.183	5.183	5.368
Cu-alloxan	−2586	6.93	−2.928	−1.517	1.411	0.706	0.708	−2.223	2.223	3.500
Fe-alloxan	−2209	11.46	−8.645	−5.207	3.438	1.719	0.291	−6.926	6.926	13.953
Ninhydrin (H_2_L^2^)	−647	5.26	−7.080	−2.765	4.315	2.158	0.232	−4.923	4.923	5.615
Cu-ninhydrin	−2593	2.52	−4.245	−2.236	2.009	1.005	0.498	−3.241	3.241	5.226
Fe-ninhydrin	−2216	2.93	−8.953	−6.195	2.758	1.379	0.363	−7.574	7.574	20.80

Generally, a small HOMO-LUMO energy gap (Δ*E*) is taken as a good indication of softness, chemical reactivity, and the complexation ability of ligands [46], Fig. 7. Ninhydrin and alloxan exhibit smaller Δ*E* values (4.315–5.004 eV) pointing to their capability to offer electrons to vacant d-orbitals of any neighboring central metal ion [47]. Also, the higher negative total energy (*E*_T_) of Cu(II) and Fe(III) complexes compared to their free ligands, Table 4, suggests the greater stability of prepared complexes [22].

In general, there is a direct correlation between biological potency and chemical reactivity indices such as dipole moment (*D*), hardness (*η*), and softness (*S*). Accordingly, the Cu-alloxan complex shows the lowest *η* (0.706 eV) and highest *S* (0.708 eV^− 1^). So, it is expected to have an adequate amount of softness and hence superior biological activity as proven practically in this research. Likewise, the estimated values of *η* (1.005 eV) and *S* (0.498 eV^− 1^) of Cu-ninhydrin predict its biological potency as confirmed later in the anticancer study section. The electrophilicity index (ω) describes the reactivity of various specific sites on the surface of the molecule, as it measures the stabilization energy when the system acquires an additional negative charge from the environment. It also quantifies the biological activity of drug-receptor interactions.

One of the validated approaches to evaluating the structure-activity relationship (SAR) is to study the electron density distribution on the surface of bioactive molecules. This allows for estimating the lipophilic capacity and hence feasible ability to penetrate the phospholipid bilayer of the cell membrane. It is well known that the cell membrane of almost all microorganisms is a phospholipid bilayer. In general, compounds of low dipole moment (*D)* are only capable of diffusing through this type of membrane as the lipophilicity is inversely proportional to the polarity degree [48]. Cu-ninhydrin has the lowest dipole moment value (2.52 Debye) and so it is expected to have good biological efficacy.

The molecular electrostatic potential (MEP) of alloxan (H_2_L^1^) and ninhydrin (H_2_L^2^) ligands were constructed theoretically to give an idea about the electronic charge distribution on their surfaces specifying the nucleophilic and electrophilic sites for possible chemical interactions, Fig. 8. The red color denotes electron-rich regions that are localized on O(15), O(16), or O(17) atoms as nucleophilic centers for chelation in the case of H_2_L^2^. This finding is in harmony with the experimental FT-IR spectral data interpretation and the suggested binding modes in this contribution, Figs. 2 and 8b. However, the blue color marks an electron deficiency zone while the neutral regions are distinguished by green color and chiefly sited on carbon atoms as in ligand H_2_L^1^, Fig. 8a.

#### Molecular Docking Study

In the recent two decades, computational simulations enhanced noticeably making it feasible to utilize computational approaches in drug design. The Molecular Operating Environmental module MOE2015 [49] software package is used to predict the biological features of candidate drugs and to anticipate the experimental results. In the present study, the protein structure of *hepatocellular carcinoma* (*2jrs*) [50] was used as the receptors docked with alloxan, ninhydrin, and their Cu(II) & Fe(III) metal complexes (inhibitors). Before the docking process, the preparation of the preprotein structure was achieved by removing water molecules and adding polar hydrogens with the MMFF94x force field. The calculated data for the docking process between all tested compounds (ligands) and the selected protein *2jrs* (receptor) are listed in Table 5; Figs. 9 and 16S, 17S.

Docking results include ligand-receptor sites, interaction type, interaction distances (Å), internal energy (E), and scoring energy (S) in kcal/mole. The negative value for energies implies the spontaneous binding of the tested inhibitor to the target protein. The data propose the best interaction stability for docked compounds. The effective ligand-receptor interaction distances were ≤ 3.5 Å in most cases, Table 5, which indicates the presence of typical real bonds and hence high binding affinity [26]. For example, the nearest interaction is observed *via* H-donors with Aspartate ASP 77 (2.62 Å) and Proline PRO 26 (2.68 Å) amino acids in the case of Fe-alloxan and Cu-ninhydrin complexes, respectively, Figs. 9 and 17S. Further, the scoring energy function (S) is taken as an indication of high ligand-protein binding affinity based on several factors such as hydrogen bonds, deformation impact, hydrophobicity, entropy, and van der Waals interaction [22]. Furthermore, five and six binding sites were observed with different amino acids in the case of Cu-ninhydrin and Fe-alloxan complexes with relatively higher negative scoring energy (− 3.9133 and − 3.9075 kcal/mol, respectively) demonstrating their high inhibition efficacy as candidates against HepG-2 cells.

**Table 5:** The docking parameters of Cu(II) and Fe(III) complexes against hepatocellular carcinoma (*2jrs*) protein

Compound	Ligand site	Receptor site	Interaction type	Distance (Å)	*E* (kcal/mol)	*S* (kcal/mol)
Alloxan	O 13	GLU 79	H-donor	2.92	–3.2	–3.8533
	N 2	LYS 83	H-acceptor	3.28	–1.3	
	O 8	LYS 83	H-acceptor	2.92	–4.5	
Ninhydrin	O 16	ASP 77	H-donor	2.87	–4.2	–3.9459
	O 17	GLY 25	H-donor	2.94	–1.5	
	O 15	GLU 79	H-acceptor	3.14	–1.8	
Cu-alloxan	O 16	ASP 77	H-donor	2.67	–7.7	–3.0909
	O 22	GLY 25	H-donor	3.08	–0.7	
	O 22	SER 76	H-donor	2.80	–2.4	
	O 8	SER 78	H-acceptor	3.11	–1.6	
	N 7	ASP 77	Ionic	3.06	–4.1	
Fe-alloxan	O 12	ALA 24	H-donor	2.78	–4.1	–3.9075
	O 16	ASP 77	H-donor	2.67	–20.7	
	O 18	ASP 77	H-donor	2.62	–25.6	
	O 7	GLU 79	H-acceptor	3.22	–2.4	
	N 6	ASP 77	Ionic	3.01	–4.4	
	N 6	ASP 77	Ionic	3.89	–0.7	
Cu-ninhydrin	O 23	SER 23	H-donor	3.01	–4.3	–3.9133
	O 24	PRO 26	H-donor	2.68	–7.6	
	O 24	MET 27	H-donor	4.41	–1.4	
	O 17	SER 23	H-acceptor	3.13	–0.8	
	6-ring	MET 27	π-H	4.28	–0.6	
Fe-ninhydrin	O 20	SER 23	H-donor	3.39	–2.1	–4.1012
	O 24	MET 27	H-donor	3.46	–7.9	
	6-ring	ARG 28	π-H	4.24	–0.6

### Biological Activity Studies

#### Antimicrobial Examination

The antimicrobial screening data of the ligands and some of their synthesized complexes were collected in Table 6; Fig. 18S. The tested samples showed negligible to good activities with mean inhibition zone values less than the used standards (*ketoconazole* for antifungal, and *Gentamycin* for antibacterial activity). The in vitro antimicrobial evaluation of the studied compounds exhibited varying inhibitory effects. Based on these data, alloxan showed no antibacterial effect against *Staphylococcus aureus* and *Bacillus subtilis*, but its iron (III) and copper (II) complexes showed a good inhibition effect against these Gram-positive bacteria. However, the Fe-alloxan complex showed the best antibacterial effect against *Escherichia coli* and *Proteus vulgaris* compared to alloxan or Cu-alloxan. Likewise, ninhydrin showed no antifungal activity against *Aspergillus fumigatus* and *Candida albicans*, but after complexation, its copper (II) complex showed good antifungal activity against both fungi strains. Also, the Cu-ninhydrin complex showed more antifungal activity (16 mm) against *Candida albicans* than Fe-ninhydrin. Similarly, Cu-ninhydrin showed more inhibition zone diameter (20 mm) against *Staphylococcus aureus* than the iron (III) complex. As both Cu(II) and Fe(III) complexes have the same M:L stoichiometry (1:1) and geometry (Oh), it appears that the metal ion type and/or the ionic radius may play an important role in enhancing or quenching the biological activities of these complexes invoking Overtone’s chelation theory [21, 51].

Seemingly, the activity in most cases is increased upon chelation where the positive charge of the metal is partially shared with donor atoms present on ligands. Further, the delocalization of p- and d- electrons over the whole chelate enhances the lipophilicity of the complex promoting its permeation into lipid membranes and blocking binding sites on the enzymes of the microorganisms [52].

**Table 6:** Antibacterial and antifungal inhibition zone in mm of ligands and some synthesized complexes

Compound	Gram-positive bacteria		Gram-negative bacteria		Fungi	
	*S. aureus*	*B. subtilis*	*P. vulgaris*	*E. coli*	*A. fumigatus*	*C. albicans*
Alloxan	NA^a^	NA	16	13	NA	NA
Fe-alloxan	13	20	20	14	NA	NA
Cu-alloxan	17	19	NA	13	NA	13
Ninhydrin	21	14	22	20	NA	NA
Fe-ninhydrin	13	NA	NA	8	NA	12
Cu-ninhydrin	20	16	20	15	15	16
Gentamycin	24	26	25	30	NT^b^	NT
Ketoconazole	NT	NT	NT	NT	17	20

^a^No activity, ^b^Not tested

#### Antioxidant Property

A lot of research effort has been focused to design new radical-scavenging compounds as effective antioxidants to control the phenomenon of oxidative stress that is produced by the imbalance between the accumulation of reactive oxygen species (ROS) in body tissues and the ability to remove potentially harmful free radicals. This accumulation of free radicals in cells can cause large chain chemical reactions and pathogenic mechanisms leading to many serious diseases such as rheumatoid arthritis, atherosclerosis, and cancer propagation [53].

The examined compounds have shown scavenging activity with different values regarding their distinct structures, compared to vitamin E as an antioxidant reference using the DPPH assay method [28], Fig. 10. Interestingly, the estimated EC_50_ value of Cu-ninhydrin complex was found to be 320 (µg/ml), which is lower than the EC_50_ of vitamin E (400 µg/ml), and hence it is expected to possess a promising antioxidant effect.

#### Anticancer Activity Assessment

The in vitro cytotoxicity activities of some synthesized metal complexes have been evaluated against a human cancer cell line (HepG-2) compared to cisplatin as a standard anticancer drug, using viability assays as described in the experimental part. The results are collected in Table 7; Figs. 11 and 12. Based on cytotoxicity results, the four examined complexes have different anticancer activity against *hepatocellular carcinoma cells* (HepG-2) according to IC_50_ values. Copper complexes of alloxan and ninhydrin are more effective against *hepatocellular carcinoma cells* than iron complexes of the same ligands. While the copper complex of ninhydrin (IC_50_ = 3.01 ± 0.3 µg/ml) is more effective against HepG-2 cells than the copper complex of alloxan (IC_50_ = 26.5 ± 1.8 µg/ml). Also, the Cu-ninhydrin complex has an even better potency impact, Fig. 12, in comparison with cisplatin (IC_50_ = 3.68 ± 0.19 µg/ml) which was used as a reference control for anticancer activity. Notably, the very low IC_50_ value for Cu-ninhydrin complex (3.01 µg/ml = 8.64 µM) is virtually under the authorization of the US NCI program that if the IC_50_ value is less than 10 µM after the 72-h incubation period, the tested compound is assumed to have in vitro promising antiproliferative action against the examined cell lines [54]. The preceding finding is in harmony to great extent with the molecular docking simulation outcomes that predicted a good propensity of Cu-ninhydrin complex to bind with the liver cancer protein (*2jrs*) as discussed earlier. So, Cu-ninhydrin complex, [Cu(HL^2^)(H_2_O)_4_].Cl could be considered a potential candidate as a chemotherapeutic agent for hepatocellular cancer.

**Table 7:** Cytotoxicity activity against HepG-2 cells of some selected complexes

Compounds	Formula	IC_50_	
	M.wt	μg/ml	μM
Fe-alloxan [Fe(HL^1^)(H_2_O)_4_].SO_4_	C_4_H_11_N_2_O_13_SFe 383.05	188 ± 6.4	> 100
Cu-alloxan [Cu(HL^1^)(H_2_O)_4_].Cl	C_4_H_11_ClN_2_O_9_Cu 330.14	26.5 ± 1.8	80.3 ± 5.4
Fe-ninhydrin [Fe(HL^2^)(H_2_O)_4_].SO_4_	C_9_H_13_O_12_SFe 401.1	231 ± 9.1	> 100
Cu-ninhydrin [Cu(HL^2^)(H_2_O)_4_].Cl	C_9_H_13_ClO_8_Cu 348.19	3.01 ± 0.30	8.64 ± 0.86
Cisplatin *Cis*-[Pt(NH_3_)_2_Cl_2_]	Cl_2_H_6_N_2_Pt 300.04	3.68 ± 0.19	12.3 ± 0.63

## Conclusions

In this research, a series of transition metal complexes of alloxan and ninhydrin were prepared and characterized by different analytical, spectroscopic, and magnetic studies. All solid complexes are of 1:1 (M:L) stoichiometry and octahedral geometry except nickel (II) complexes exist in a 4-coordinate structure. Density functional theory (DFT) is used to construct the optimized structures of the ligands and their molecular electrostatic potential surfaces. Further, the two ligands and four of the designed complexes were subjected to molecular docking simulation as a preliminary SAR study. Biological screening of some compounds was tested as antioxidant and antimicrobial (against four gram-positive & gram-negative bacteria, and two fungi strains). Compounds with noticeable activity may be considered as a starting point for the development of some new therapeutic drugs. Some examined metal complexes display anticancer activity against *hepatocellular carcinoma cells* (HepG-2) but to different degrees. According to the IC_50_ values, the Cu-ninhydrin complex has a better potency impact compared with cisplatin. This is in good correlation with the molecular docking simulation outcomes that predicted a strong binding of Cu-ninhydrin complex to the liver cancer protein (*2jrs)*. So, the Cu-ninhydrin complex could be considered a potential candidate as a chemotherapeutic agent for *hepatocellular* cancer after further clinical studies such as ADMET-score, intracellular ROS generation, clinical trials, and drug approval processes. Besides, the copper (II) complex derived from ninhydrin is economically expected to be of low cost to other platinum-based chemical drugs.

## Electronic Supplementary Material

Below is the link to the electronic supplementary material.

Supplementary Material 1

## Data Availability

Supplementary data associated with this article can be found in the online version.
