# Supplementary material for: Synthesis, Physicochemical Characterization, Biological Assessment, and Molecular Docking Study of Some Metal Complexes of Alloxan and Ninhydrin as Alterdentate Ligands
Source: J Inorg Organomet Polym Mater. 2023 Apr 30:1–18. Online ahead of print. doi: 10.1007/s10904-023-02661-5 (PMC10149045; doi:10.1007/s10904-023-02661-5)
Supplement: Supplementary file 1 — Supplementary Material 1 [file 10904_2023_2661_MOESM1_ESM.docx]

Supplementary data

**Synthesis, physicochemical characterization, biological assessment, and molecular docking study of some metal complexes of alloxan and ninhydrin as alterdentate ligands**

**Mamdouh S. Masoud^1^ . Galila A. Yacout^2^ . Bassant A. Abd-El-Khalek^1^ . Ahmed M. Ramadan^1^***

^1^ Chemistry Department, Faculty of Science, Alexandria University, P.O. Box 426, Alexandria 21321, Egypt

^2^ Biochemistry Department, Faculty of Science, Alexandria University, P.O. Box 21511, Alexandria, Egypt

* Corresponding author: A. M. Ramadan, [ahmed.m.ramadan@alexu.edu.eg](mailto:ahmed.m.ramadan@alexu.edu.eg)

**
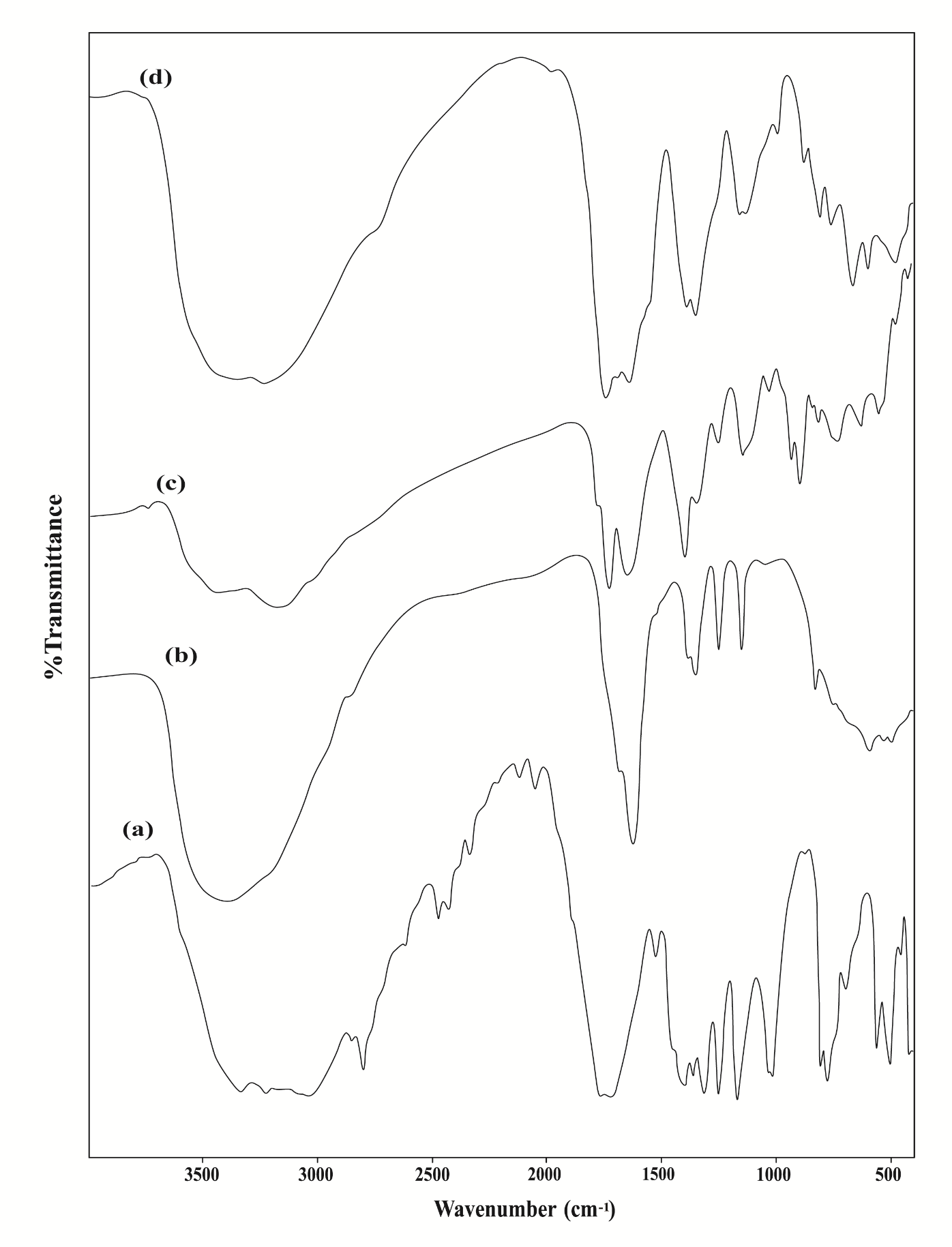
**

**Fig. 1S** FT-IR of a) alloxan and its b) Cu, c) Mo, d) Zr complexes


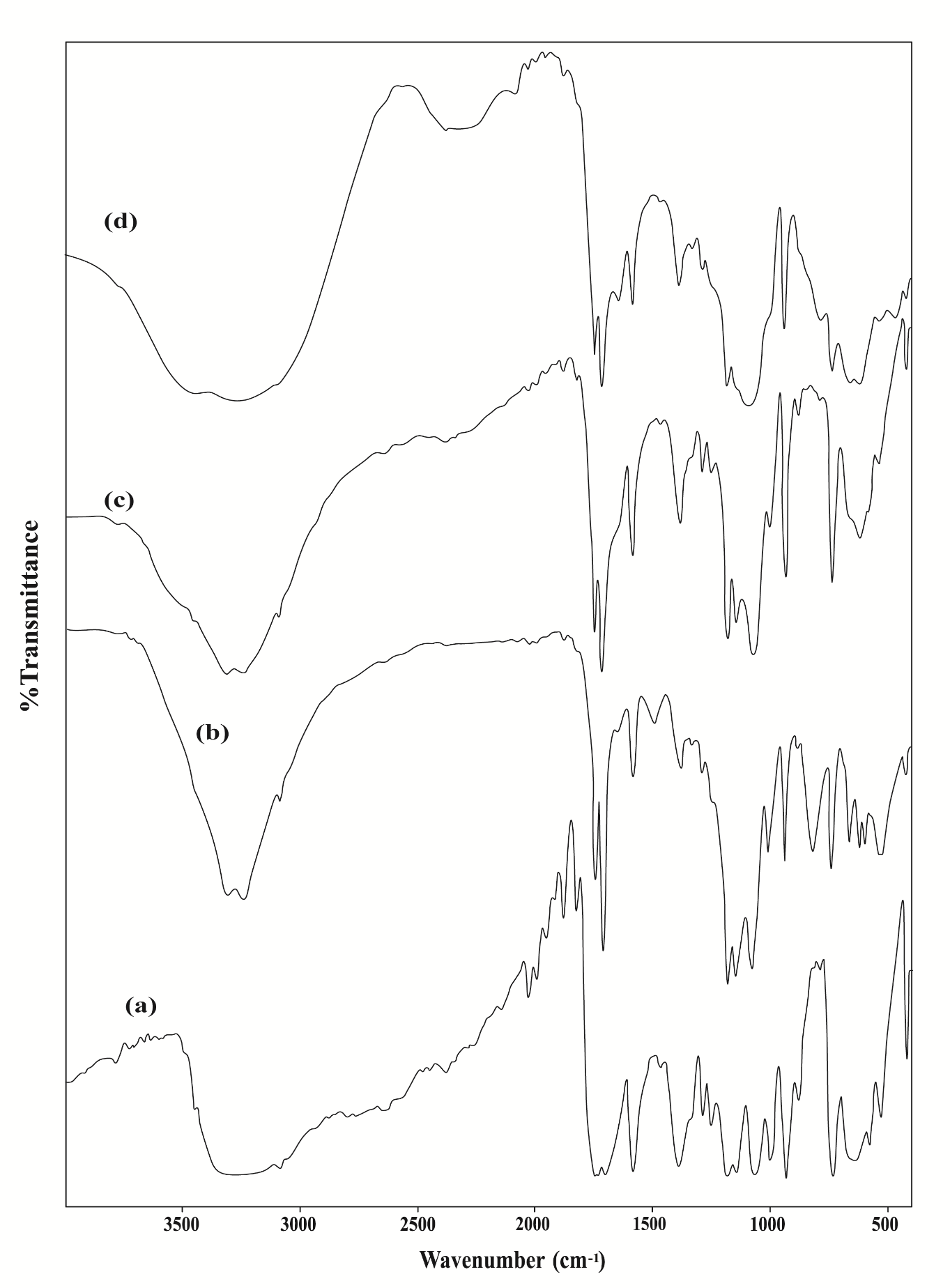


**Fig. 2S** FT-IR of a) ninhydrin and its b) Fe, c) Co, d) Ni complexes

**Fig. 3S** FT-IR of (a) ninhydrin, and its b) Cu, c) Mo, d) Zr complexes


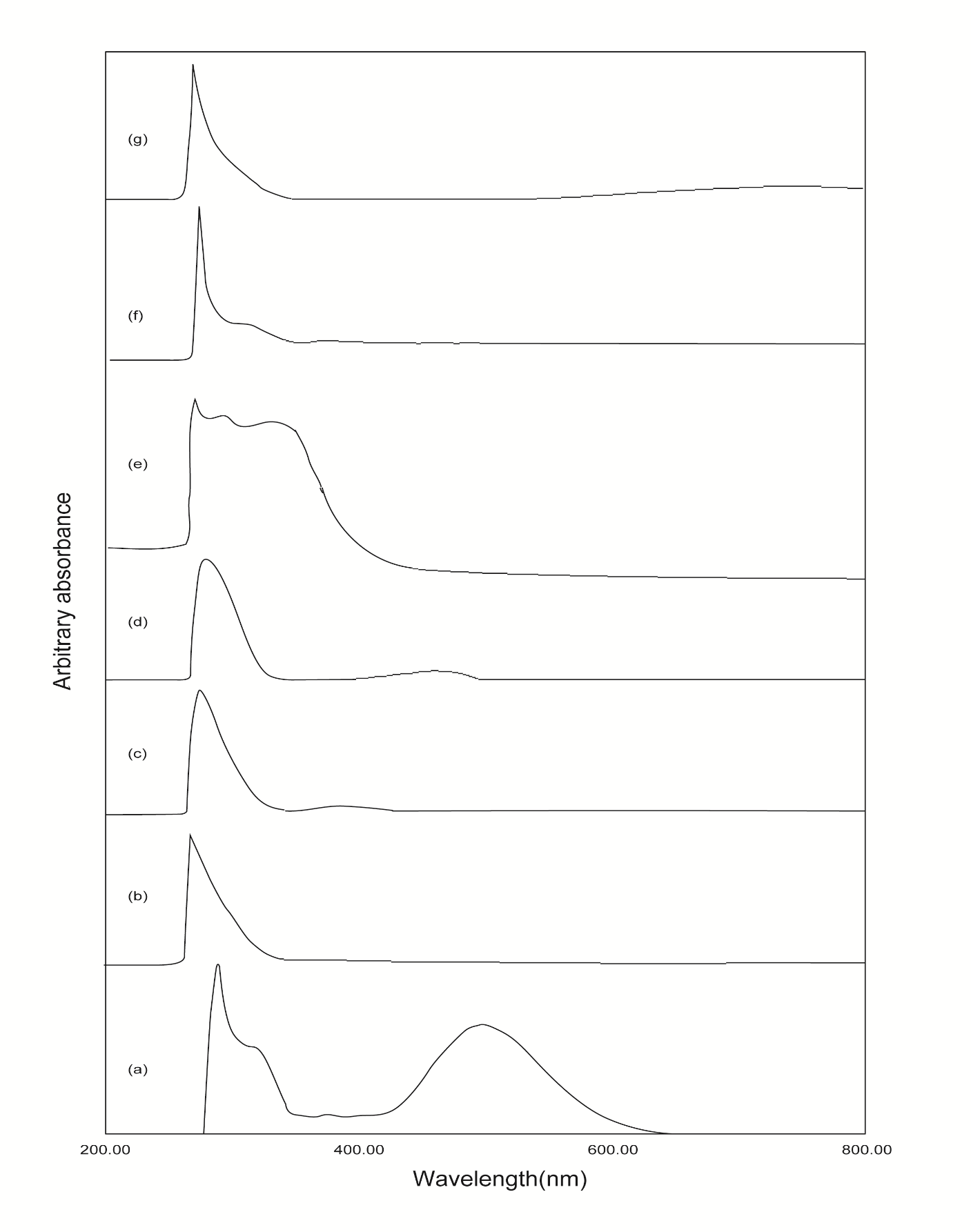


**Fig. 4S** Nujol mull electronic spectra of a) Alloxan b) Fe-alloxan c) Co-alloxan d) Ni-alloxan e) Cu-alloxan f) Zr-alloxan g) Mo-alloxan complexes


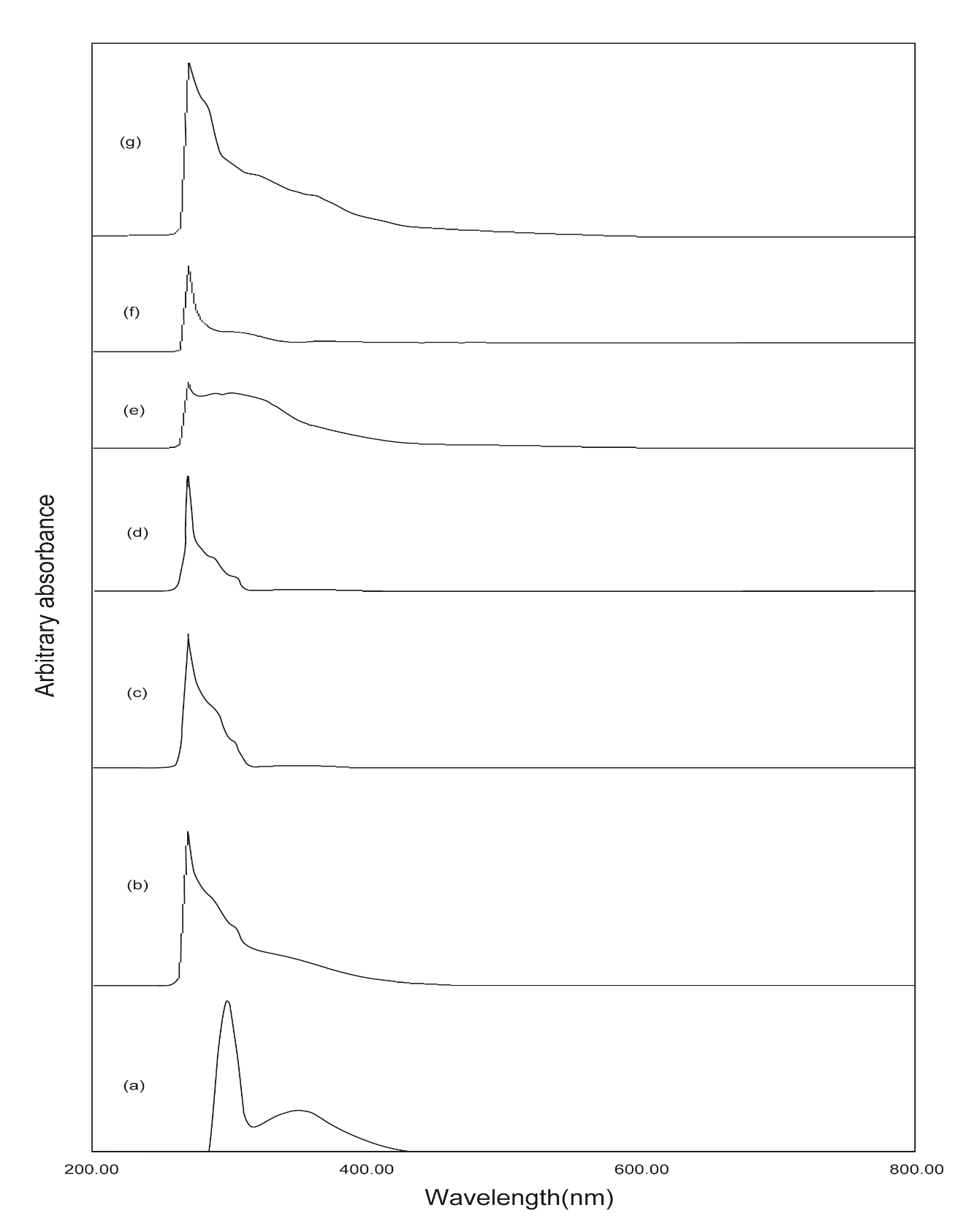


**Fig. 5S** Nujol mull electronic spectra of a) Ninhydrin b) Fe-ninhydrin c) Co-ninhydrin d) Ni-ninhydrin e) Cu-ninhydrin f) Zr-ninhydrin g) Mo-ninhydrin complexes

**Fig. 6S** Suggested structures of inner sphere metal alloxan complexes


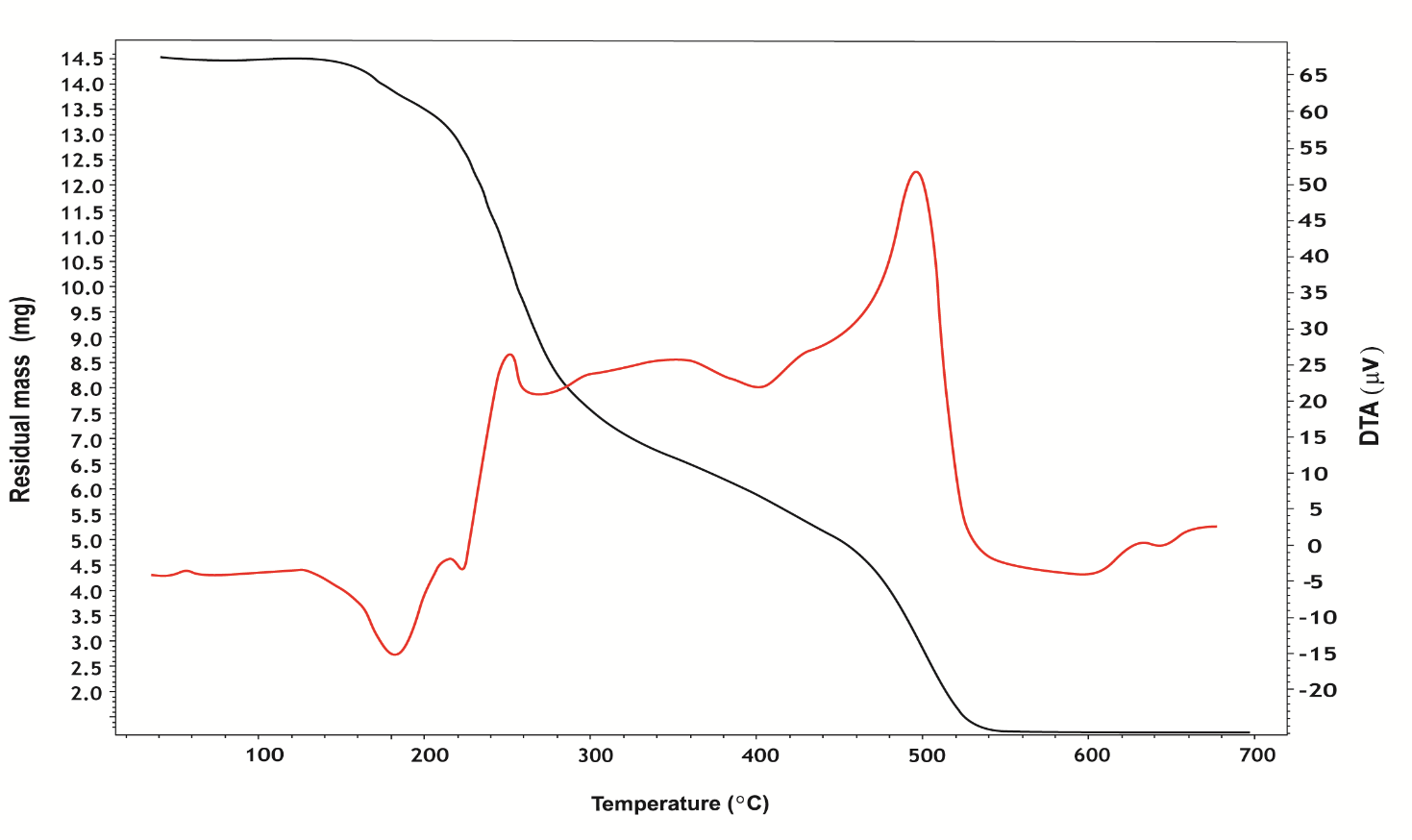


**Fig. 7S** TGA and DTA curves of alloxan


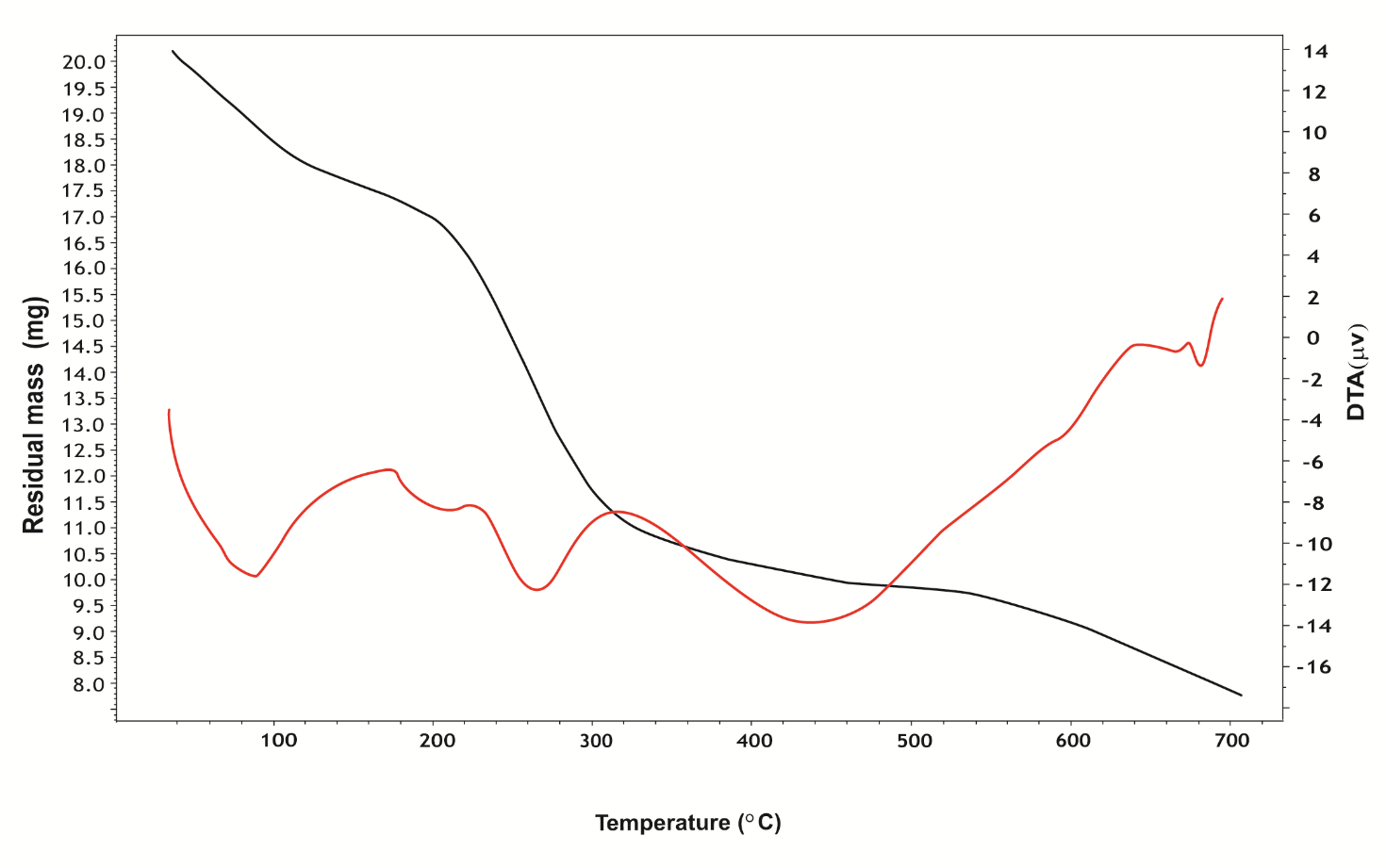


**Fig. 8S** TGA and DTA curves of Zr-alloxan complex


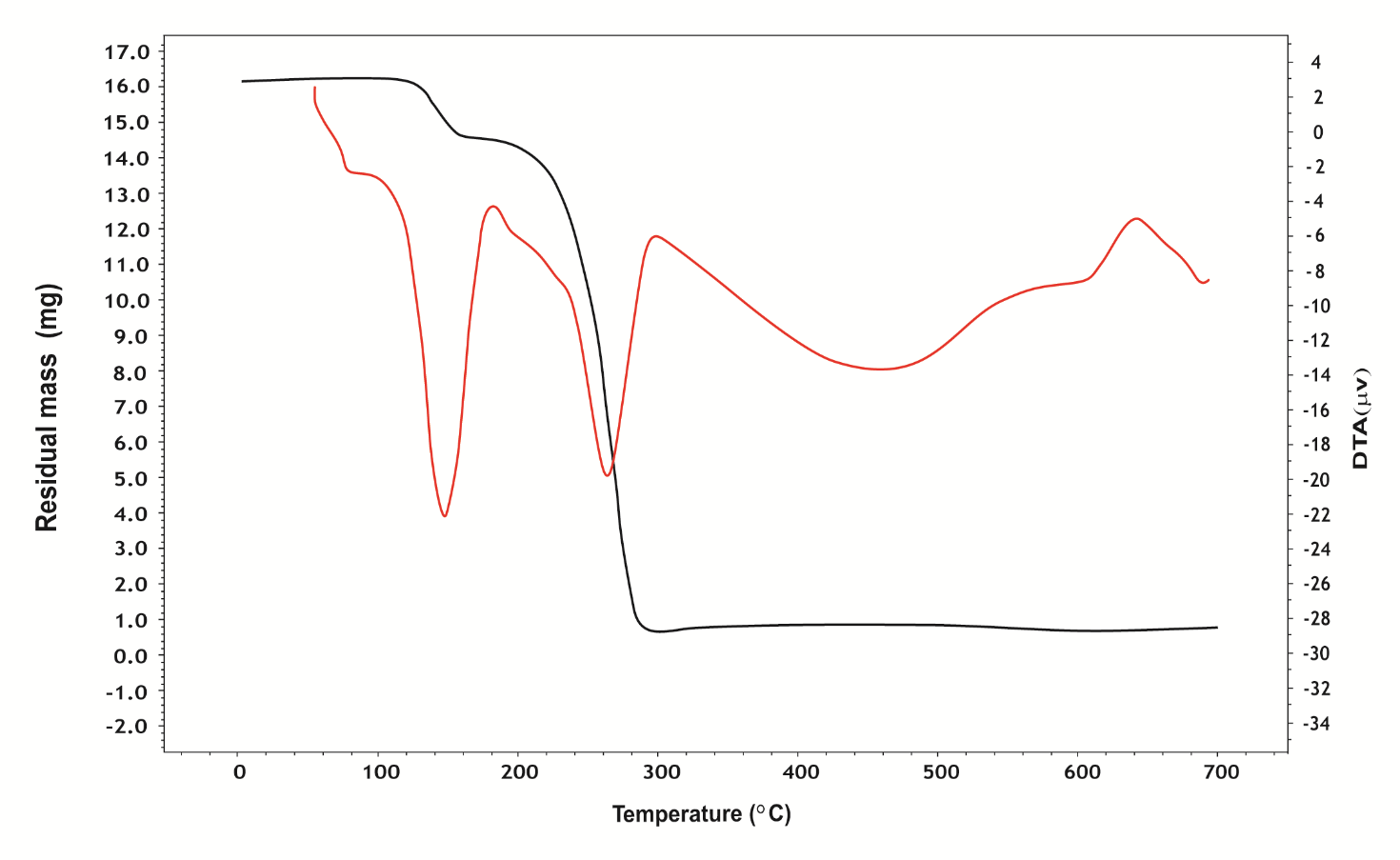


**Fig. 9S** TGA and DTA curves of ninhydrin


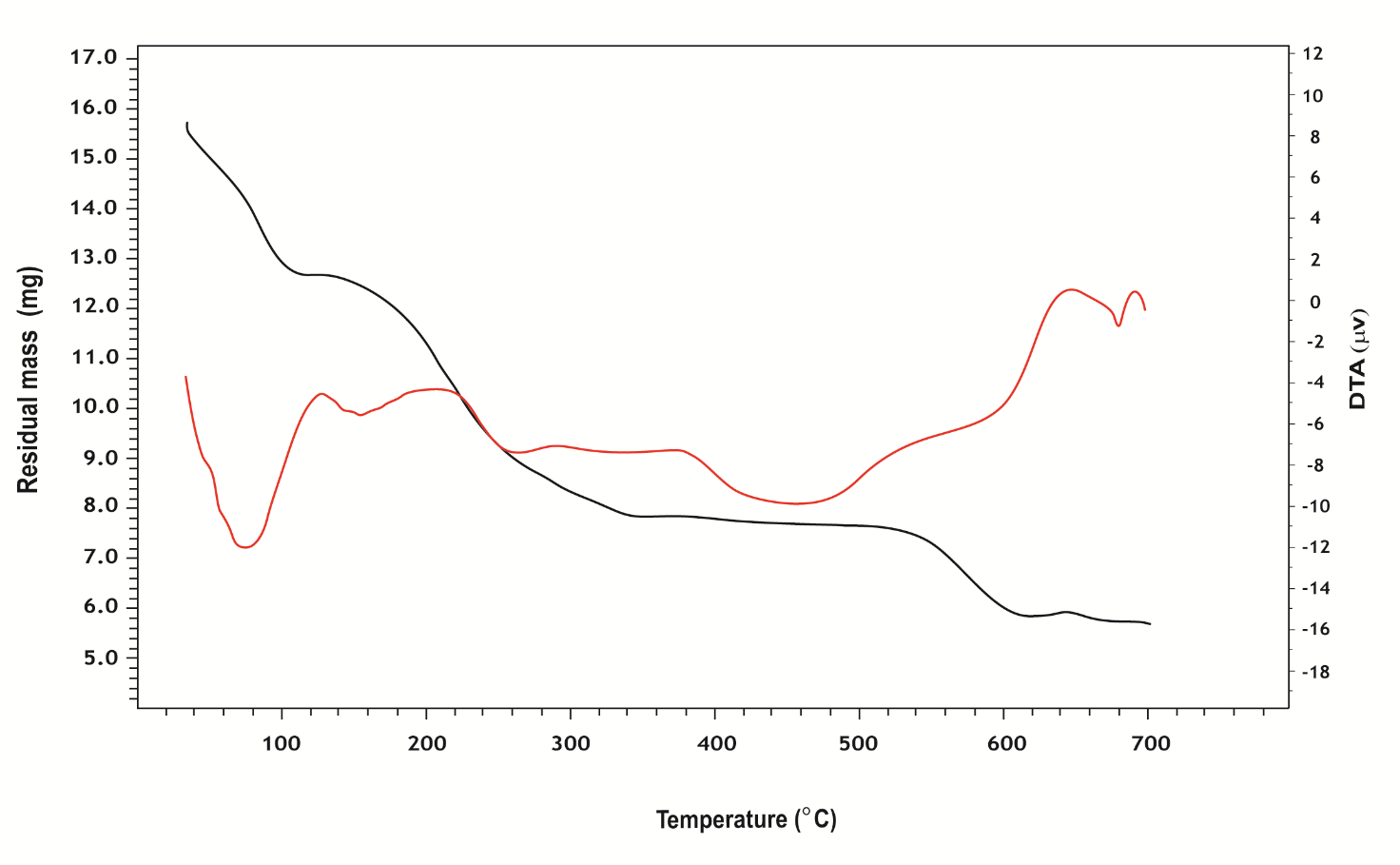


**Fig. 10S** TGA and DTA curves **of** Cu-ninhydrin complex


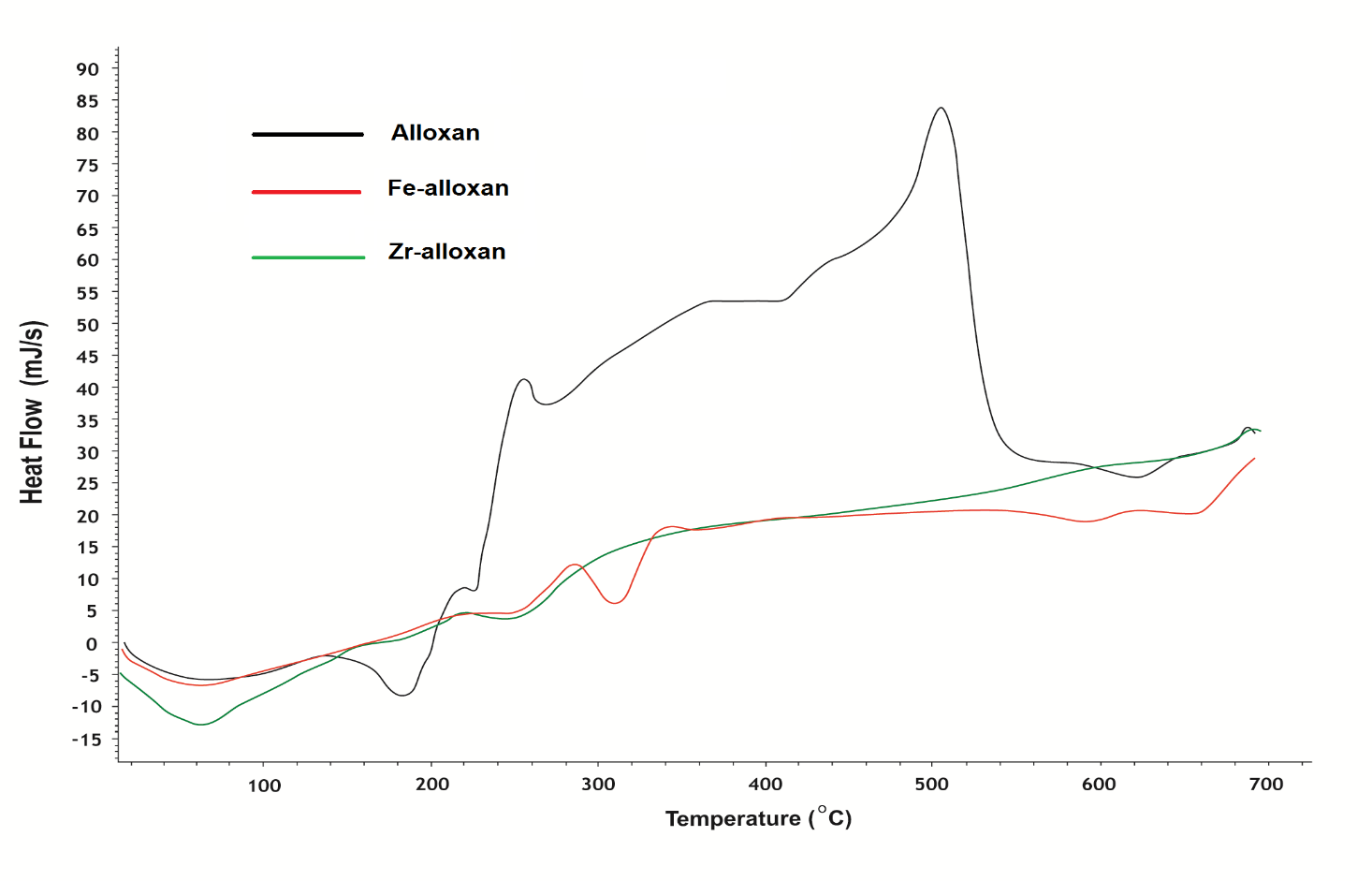


**Fig. 11S** DSC curves of ninhydrin and its Cu and Mo complexes

**
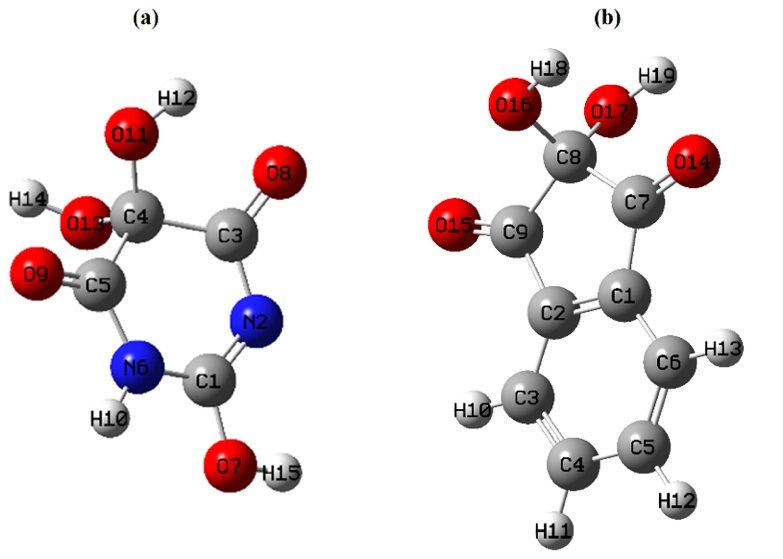
**

**Fig. 12S** Optimized structures of **(**a) alloxan, (b) ninhydrin using DFT-B3LYP/ 6-31G method

**
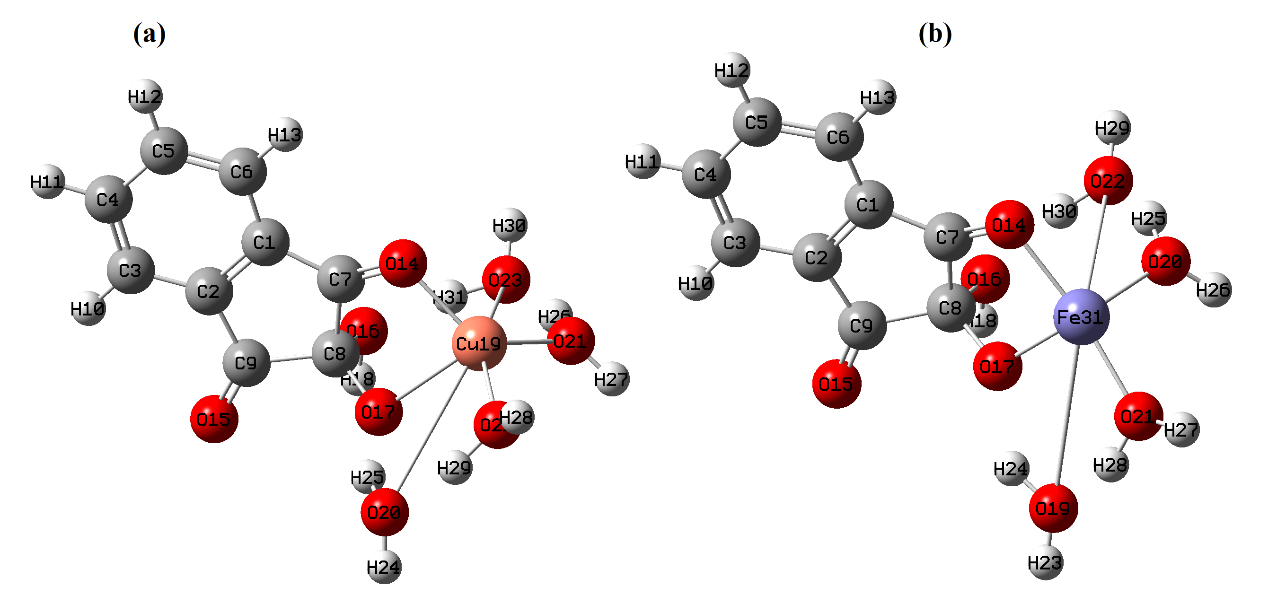
**

**Fig. 13S** Optimized structures of (a) Cu-ninhydrin and (b) Fe-ninhydrin using DFT-B3LYP/ 6-31G method

**
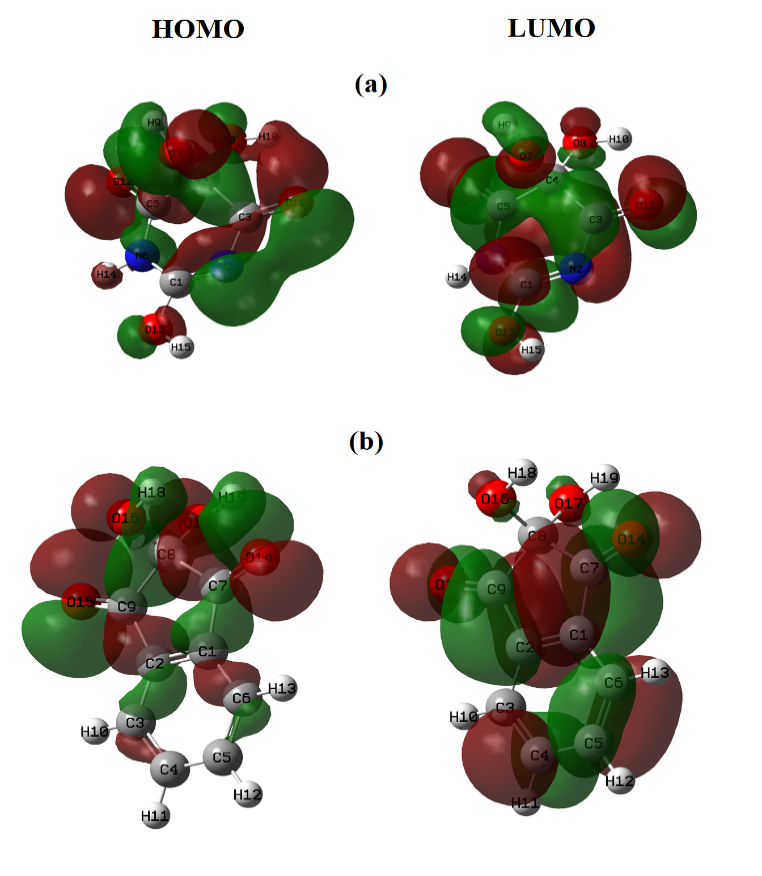
**

**Fig. 14S** LUMO and HOMO of the ligands (a) alloxan and (b) ninhydrin

_
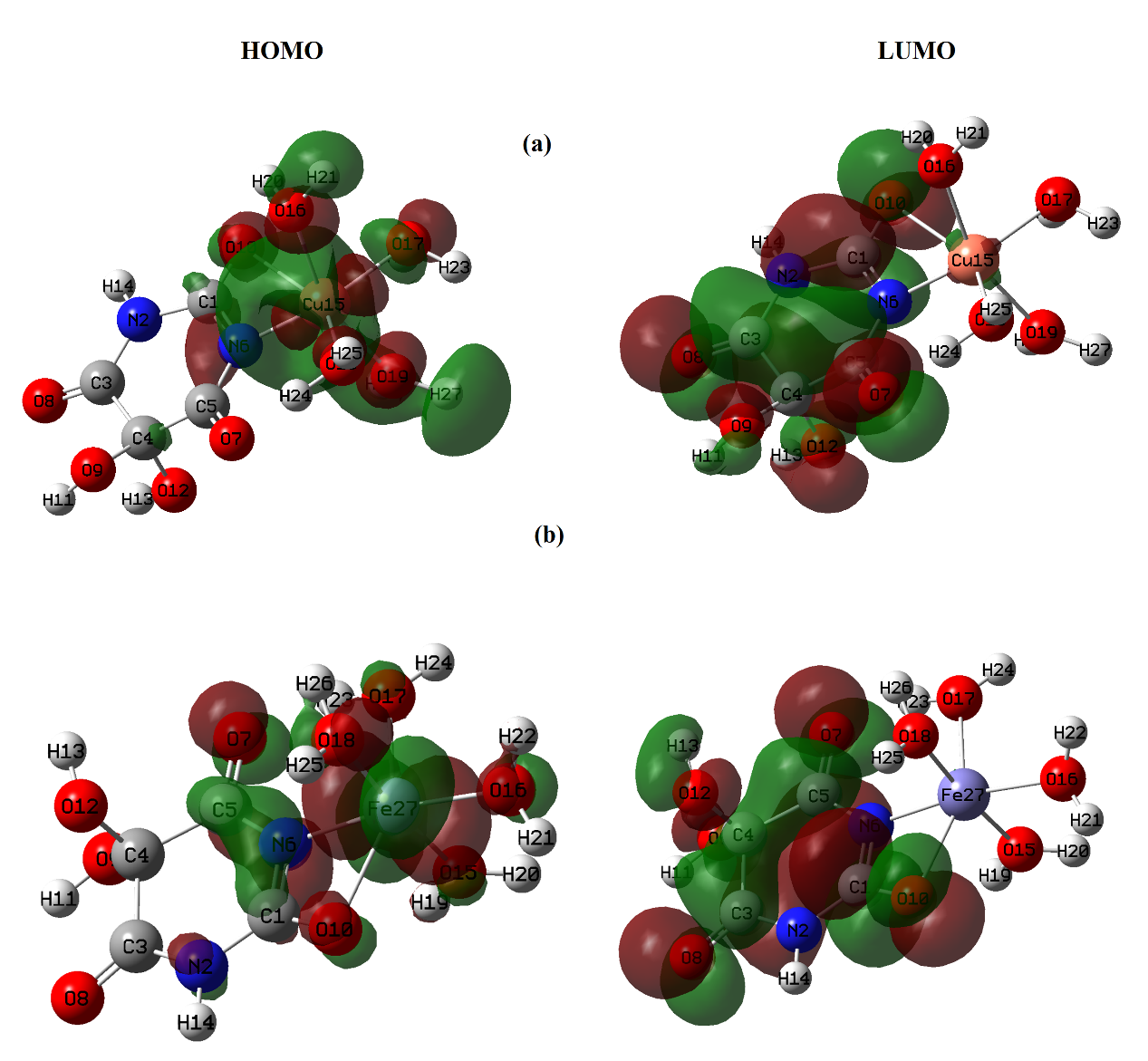
_

**Fig. 15S** LUMO and HOMO of (a) Cu-alloxan and (b) Fe-alloxan


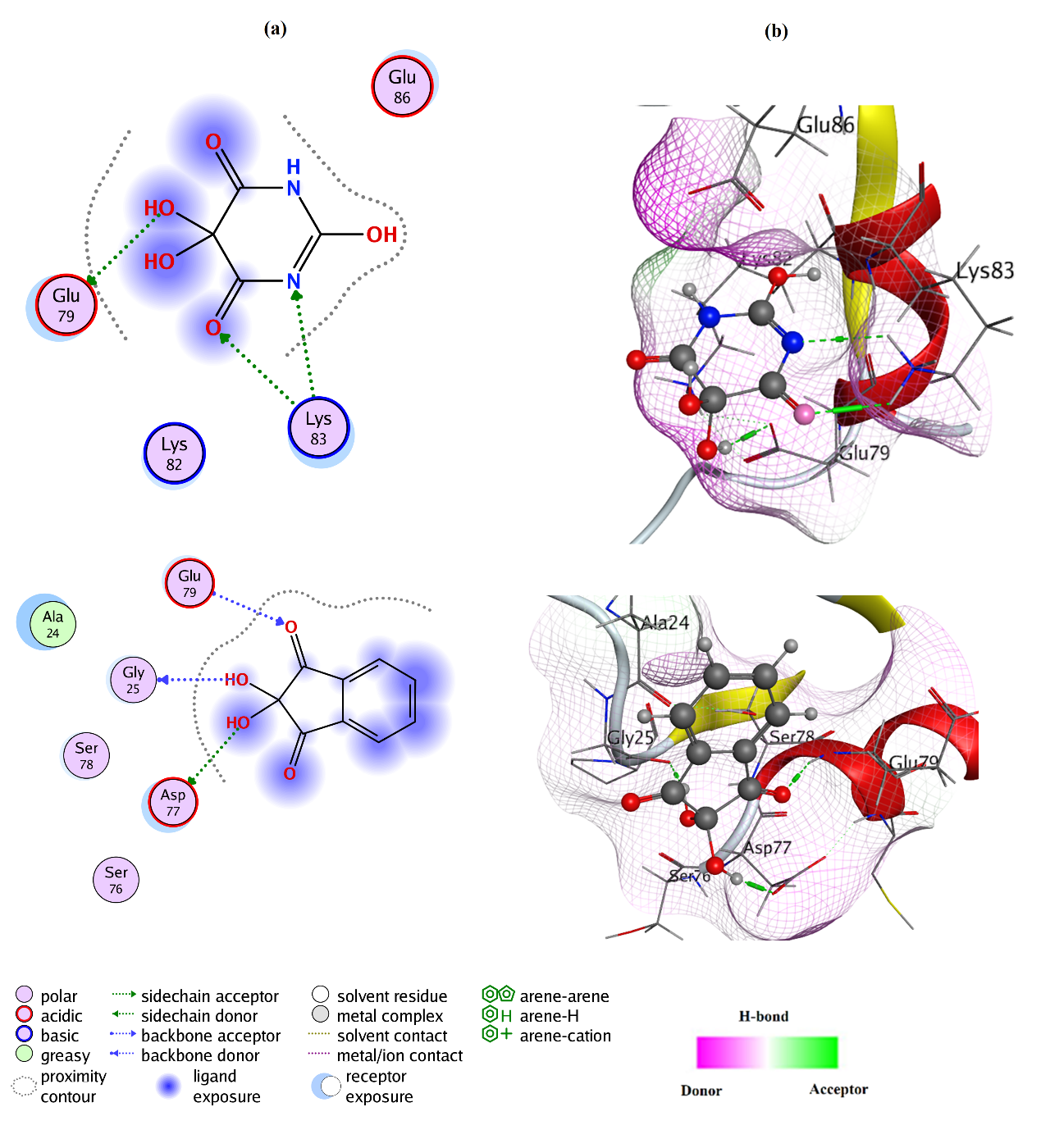


**Fig. 16S** Ligand- receptor interactions (a) and receptor lipophilicity maps (b) of the best-docked poses of alloxan and ninhydrin ligands


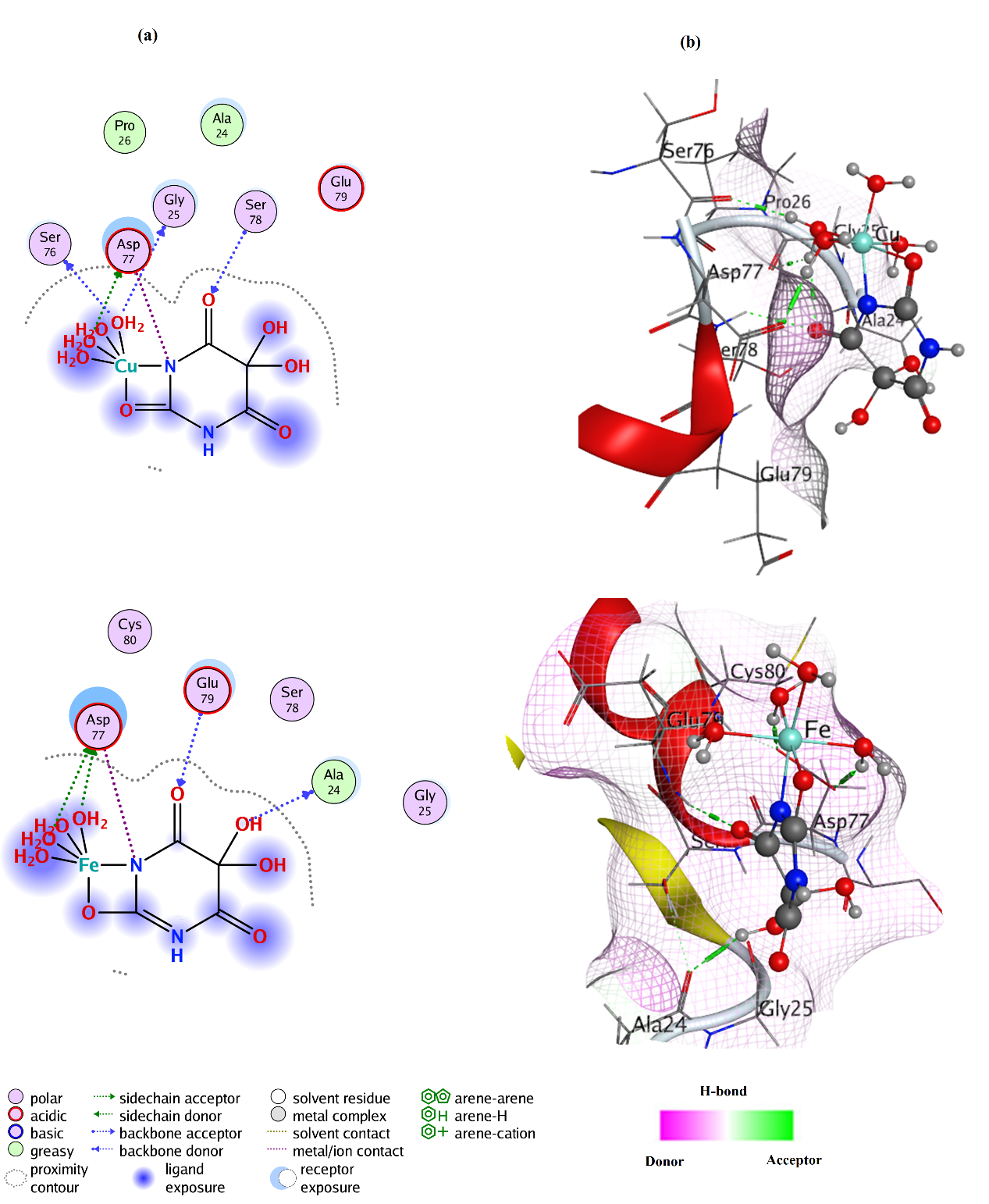


**Fig. 17S** Ligand- receptor interactions (a) and receptor lipophilicity maps (b) of the best-docked poses of Cu-alloxan and Fe-alloxan complexes

| ***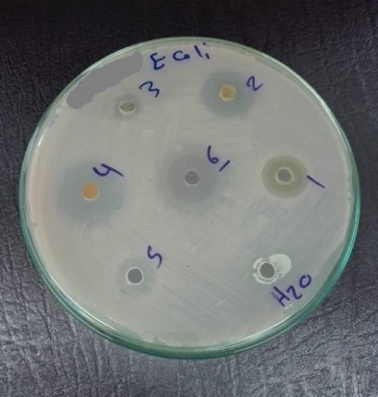***  **(a)** | ***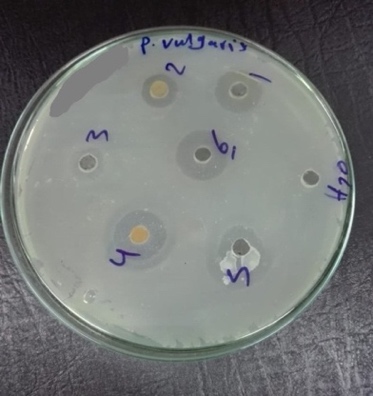***  **(b)** |
| --- | --- |
| ***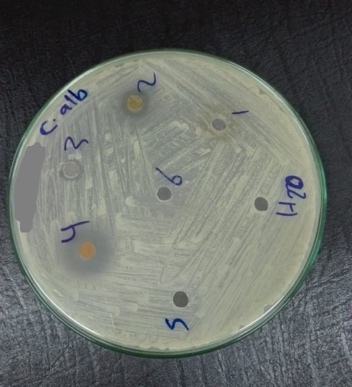***  **(c)** | ***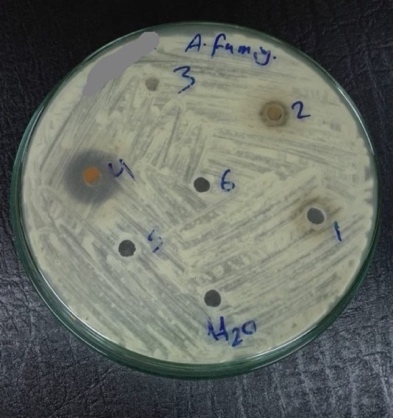***  **(d)** |
| ***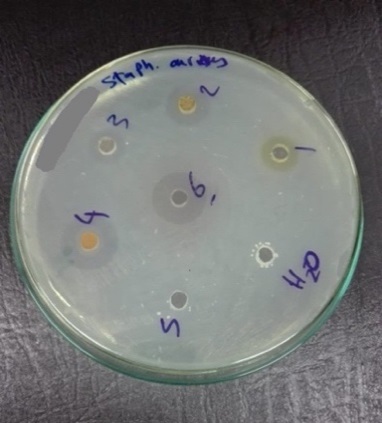***  **(e)** | ***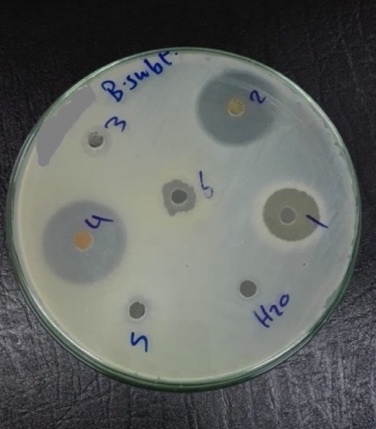***  **(f)** |

**Fig. 18S** Mean zone of inhibition for all examined compounds in case of: (a) Escherichia coli (b) Proteus vulgaris (c) Candida albicans (d) Aspergillus fumigatus (e) Staphylococcus aureus (f) Bacillus subtilis; Sample code: 1: Fe-alloxan 2: Cu-alloxan. 3: Fe-ninhydrin 4: Cu-ninhydrin 5: Alloxan 6: Ninhydrin

**Table 1S** Fundamental infrared bands of alloxan and its metal complexes

| **Assignment** | **Zr-L^1^** | **Mo-L^1^** | **Cu-L^1^** | **Ni-L^1^** | **Co-L^1^** | **Fe-L^1^** | **H_2_L^1^** |
| --- | --- | --- | --- | --- | --- | --- | --- |
| ν(OH) & ν(NH)  overlapping | 3348(b)  3232(b) | 3445(b)  3178(b) | 3401(b)  3168(b) | 3448(b)  3041(b) | 3329(b)  3267(b) | 3347(b)  3046(b) | 3340  3042 |
| ν(C=O) | 1741  1696(sh) | 1732  1654 | 1722(sh)  1686(sh) | 1730  1650 | 1738  1708 | 1757  1722 | 1767  1715 |
| ν(N=C-O) | 1639 | 1630(sh) | 1625 | …… | …… | 1633 | …… |
| δ(NH) | 1388 | 1402 | 1383 | 1423 | 1424 | 1419 | 1449 |
| δ(OH) | 1347 | 1347 | 1352 | 1350 | 1375 | 1397 | 1399 |
| ν(C-N) | 1157 | 1148 | 1148 | 1112(sh) | 1155 | 1170 | 1170 |
| δ(C=O) | 808 | 818 | 830 | 802 | 808 | 806 | 804 |
| δ(NH) | 757 | 738 | 742(sh) | 757 | 775 | 785 | 776 |
| ν(M-O) | 600 | 636 | 595 | 624 | 606 | 624 | …. |
| ν(M-N) | 482 | 481 | 498 | 494 | 501 | 484 | …. |

**Table 2S** Fundamental infrared bands of ninhydrin and its metal complexes

| **Assignment** | **Zr-L^1^** | **Mo-L^1^** | **Cu-L^1^** | **Ni-L^2^** | **Co-L^2^** | **Fe-L^2^** | **H_2_L^2^** |
| --- | --- | --- | --- | --- | --- | --- | --- |
| ν(OH) | 3354(b) | 3416(b)  3158 | 3411(b) | 3489  3245 | 3313  3242 | 3311  3244 | 3300(b) |
| ν(C-H) | 3048(sh) | 3041(sh) | 3096(b) | 3045 | 3087 | 3089 | 3086 |
| ν(C=O) | 1756 | 1761 | 1748(sh) | 1748 | 1749 | 1749 | 1749 |
| ν(C=C) | 1720  1605 | 1665(b) | 1628(b) | 1717  1589 | 1717  1588 | 1718  1590 | 1703  1588 |
| δ(OH) | 1465(w) | 1486(w) | 1486(w) | 1485(w) | 1492(w) | 1497 | 1470 |
| ν(C-C) | 1418 | 1403 | 1412 | 1388 | 1388 | 1387 | 1389 |
| δ(C-H) | 740 | 725 | 763 | 740 | 740 | 741 | 740 |
| ν(M-O) | 483 | 487 | 520 | 472 | 488(sh) | 528 | …. |

**Table 3S** Kinetic parameters for ligands and some of their complexes

| **∆*G** (kJ/mol)** | **∆*S** (kJ/mol.K)** | **∆*H** (kJ/mol)** | **E_a_ (kJ/mol)** | **Peak Temp.(K)** | **Peak** | **Compound** |
| --- | --- | --- | --- | --- | --- | --- |
| 139.3 | -0.23 | 31.9 | 35.7 | 457 | 1^st^ | Alloxan  **C_4_H_4_N_2_O_5_** |
| 253.5 | -0.24 | 63.63 | 70.1 | 778 | 2^nd^ |  |
| 125.6 | -0.23 | 24.1 | 27.6 | 433 | 1^st^ | Fe-alloxan  **[Fe(HL^1^)(H_2_O)_4_].SO_4_** |
| 328.8 | -0.10 | 255.8 | 261.8 | 716 | 2^nd^ |  |
| 273.5 | -0.11 | 177.0 | 184.3 | 877 | 3^rd^ |  |
| 24.2 | -0.01 | 18.8 | 23.3 | 537 | 1^st^ | Zr-alloxan  **[Zr(HL^1^)Cl_3_(H_2_O)]** |
| 25.8 | -0.01 | 18.5 | 24.5 | 727 | 2^nd^ |  |
| 204.8 | -0.11 | 163.2 | 166.7 | 420 | 1^st^ | Ninhydrin  **C_9_H_6_O_4_** |
| 291 | -0.23 | 127.4 | 133.4 | 716 | 2^nd^ |  |
| 77.1 | -0.12 | 24.5 | 28.1 | 431 | 1^st^ | Cu-ninhydrin  **[Cu(HL^2^)(H_2_O)_4_].Cl** |
| 122.2 | -0.14 | 47.9 | 52.3 | 532 | 2^nd^ |  |
| 177.4 | -0.13 | 80.8 | 86.8 | 723 | 3^rd^ |  |
| 121.2 | -0.26 | 28.2 | 31.2 | 363 | 1^st^ | Mo-ninhydrin  **[MoO_2_(HL^2^)(H_2_O)OH]** |
| 202.1 | -0.10 | 149.4 | 153.6 | 506 | 2^nd^ |  |
| 185.3 | -0.20 | 68.1 | 74.1 | 722 | 3^rd^ |  |

**Table 4S** Thermal transitions of ligands and some of their complexes

| **T_m (_ºc)** | **T_c_ (ºc)** | **T_g_ (ºc)** | **Compound** |
| --- | --- | --- | --- |
| 254.7 | 183.9 | …… | Alloxan  **C_4_H_4_N_2_O_5_** |
| 344.5 | 242.3 | 143.2 | Fe-alloxan  **[Fe(HL^1^)(H_2_O)_4_].SO_4_** |
| 266.2 | 190.2 | 128.4 | Zr-alloxan  **[Zr(HL^1^)Cl_3_(H_2_O)]** |
| 262.0 | 147.0 | …… | Ninhydrin  **C_9_H_6_O_4_** |
| 381.1 | 247.2 | 128.6 | Cu-ninhydrin  **[Cu(HL^2^)(H_2_O)_4_].Cl** |
| 450.0 | 229.2 | 137.0 | Mo-ninhydrin  **[MoO_2_(HL^2^)(H_2_O)OH]** |
